# Toward Sustainable Lithium Recovery: A Universal Hydrothermal Approach for Lithium Extraction

**DOI:** 10.1021/acsami.6c02211

**Published:** 2026-04-10

**Authors:** Zexin Wang, Jiahui Hou, Zifei Meng, Jinzhao Fu, Zeyi Yao, Zhenzhen Yang, Yan Wang

**Affiliations:** † Department of Mechanical and Materials Engineering, 8718 Worcester Polytechnic Institute, 100 Institute Road, Worcester, Massachusetts 01609, United States; ‡ Chemical Sciences & Engineering Division, Argonne National Laboratory, 9700 S Cass Ave, Lemont, Illinois 60439, United States

**Keywords:** lithium-ion batteries recycling, hydrothermal reaction, high selectivity, ultrahigh efficiency, universal feedstock, battery-grade product

## Abstract

The rapid growth of lithium-ion battery (LIB) deployment presents critical challenges in sustainable end-of-life management and raw material recovery. Conventional pyrometallurgical and hydrometallurgical methods suffer from high energy demand, lithium loss, and complex wastewater treatment. This study established a universal, highly efficient, and sustainable hydrothermal route for lithium extraction and material recovery from various spent lithium-ion battery cathodes using 1,2,4,5-benzenetetracarboxylic acid (BTCA). The optimized process achieved over 99% lithium leaching efficiency for lithium iron phosphate (LFP) and LiNi_*x*_Mn_*y*_Co_1–*x–*_ *_y_* O_2_ (NMC), with transition metal coleaching below 1%. It was also broadly applicable to lithium manganese oxide, lithium cobalt oxide, and black mass, achieving 98.5%, 98.95%, and 94.06% leaching efficiencies, respectively. The extracted lithium was directly converted into battery-grade lithium sources, while transition metals were recovered as oxides. Unreacted BTCA was efficiently regenerated and reused without degradation. Electrochemical evaluation confirmed that cathode materials synthesized with recovered lithium exhibit comparable performance to commercial products. Compared to conventional hydrometallurgy, the BTCA-based process increased revenue by over 40% and reduced greenhouse gas emissions by up to 39%. This closed-loop, chemistry-agnostic strategy offered a scalable and economically viable solution for industrial LIB recycling, enabling resource circularity and reducing dependency on primary critical materials.

## Graphical abstract

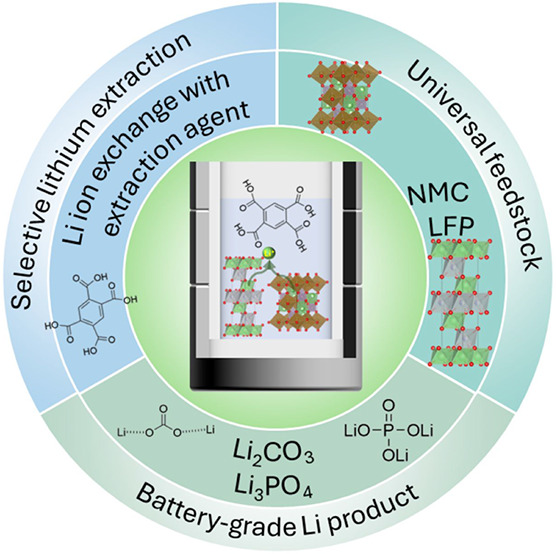

## 1 Introduction

The widespread deployment of lithium-ion batteries (LIBs) in electric vehicles, grid storage systems, and portable electronics has led to a growing accumulation of spent batteries, posing both environmental hazards and resource recovery opportunities.^1,2^ Cathode materials, which account for the largest fraction of critical metals in LIBs, are of particular interest for recycling.^3^ Among them, LiNi_*x*_Mn_*y*_Co_1–*x–*_ *_y_* O_2_ (NMC) and lithium iron phosphate (LiFePO_4_, LFP) are two dominant chemistries with contrasting structural, chemical, and electrochemical characteristics that influence their end-of-life recycling strategies.^4^ 360,000 tons of LIBs will be decommissioned from electronic devices and electric vehicles by 2025; this number is expected to grow rapidly to 1.22 million tons in 2030.^5^ These challenges underscore the urgency of developing efficient, sustainable, and economically viable recycling methods to manage spent battery materials, mitigate environmental pollution, and recover valuable battery-grade raw materials. Notably, spent LFP batteries contain up to 5 wt % of lithium, markedly higher than the lithium concentrations typically found in natural lithium ores, which range from 0.2 to 1.5 wt %.^6,7^ Similarly, Ni, Mn, and Co minerals are unevenly distributed around the world and are high-value elements.^4^ Given the uneven geographic distribution, scarcity, and geopolitical concerns associated with raw materials for lithium-ion batteries, recycling end-of-life lithium-ion batteries emerges as a strategic approach to offset lithium shortages, stabilize the lithium supply chain, and promote sustainable resource utilization.^8–11^

Currently, spent lithium-ion battery recycling methodologies predominantly fall into pyrometallurgical, hydrometallurgical, and direct recycling/upcycling approaches.^12,13^ The traditional pyrometallurgy approach involves thermal treatment, including roasting or smelting, conducted at elevated temperatures, which often exceed 800–1,000 °C, aiming to convert metal oxides to their elemental forms or simpler oxides for subsequent recovery.^14,15^ This method is favored industrially due to its straightforwardness, adaptability to different battery chemistries, and ease of scalability.^16^ However, pyrometallurgy encounters significant difficulties when applied to LFP cathodes due to their inherently stable olivine structure, characterized by strong P–O and Fe–O bonds.^17^ This structural stability prevents the complete reduction of iron phosphates, resulting in relatively low lithium recovery efficiencies and necessitating extremely high operational temperature, which dramatically escalates energy consumption and associated carbon dioxide emissions.^18^ Furthermore, the generation of hazardous gases poses additional environmental and health hazards.^19^ These substantial disadvantages render pyrometallurgical recycling environmentally unsustainable and economically unfavorable for LFP and NMC cathode materials.^20,21^

Alternatively, hydrometallurgy recycling processes involve selective extraction of valuable materials from the cathode materials using chemical leaching techniques, typically employing strong inorganic acids, such as sulfuric acid, hydrochloric acid, or nitric acid.^22–24^ Although the leaching efficiency of strong inorganic acids is high, their use typically involves substantial acid and alkali consumption, leading to increased waste liquid generation. Moreover, these acids have notable disadvantages, such as intensive chemical usage, severe corrosion of equipment, and enhanced secondary environmental pollution. Additionally, harmful gases like SO_3_, Cl_2_, and NO_*x*_ are generated, posing further environmental risks.^25,26^

Recognizing these limitations, recent research efforts have prioritized developing novel organic acid–based leaching approaches with enhanced selectivity, efficiency, and sustainability. Therefore, researchers began using organic acids instead of inorganic acids. However, many organic-acid leaching systems exhibit weaker acidity and slower kinetics than strong mineral acids, and industrial hydrometallurgy therefore often employs redox additives (e.g., H_2_O_2_) to tune transition-metal dissolution and improve overall leaching performance. While organic acids offer a more environmentally benign alternative to inorganic acids, their widespread application is hindered by high reagent costs and weaker leaching capability, necessitating the use of auxiliary reducing agents and complicating efforts to reduce overall process costs.^27^ Ali et al. developed a lithium recovery process using succinic acid for the selective leaching of Li from spent LFP cathode materials. While the method demonstrated moderate selectivity, the addition of an oxidizing agent such as H_2_O_2_ was required, resulting in over 5% coleaching of Fe and P, which introduces additional costs associated with oxidant consumption and downstream purification requirements.^28^ Similarly, Punt et al. proposed a citric acid–based leaching process with the aid of an oxidant for lithium recovery from NMC cathodes.^29^ Although effective in extracting lithium, this approach exhibited poor selectivity, with simultaneous dissolution of transition metals such as Ni, Co, and Mn. Consequently, stringent separation and purification steps are necessary to isolate battery-grade lithium carbonate from the complex leachate. Both methods are limited in their applicability to specific cathode chemistries, succinic acid is suitable for LFP, while citric acid is primarily used for layered NMC-type materials. This lack of universality presents a major challenge for the practical recycling of mixed black mass, which typically contains multiple cathode chemistry.^28,30–37^ In parallel, deep eutectic solvents (DESs) have been widely investigated for metal extraction from spent lithium-ion battery black mass, offering tunable coordination environments and the potential for selective metal dissolution.^38–41^ Relative to many reported DES-based systems, the present BTCA-based hydrothermal process emphasizes faster lithium extraction kinetics, higher lithium selectivity across diverse cathode chemistries, and a leaching agent that can be readily recovered and reused through a straightforward regeneration pathway. Microwave-assisted water leaching has been reported as an effective approach for lithium recovery from cathode materials.^40–43^ Microwave irradiation promotes rapid carbothermic reduction and facilitates the formation of water-soluble lithium species. Notably, Cornelio et al. achieved lithium recoveries exceeding 90% from NMC black mass.^44^ By comparison, the present BTCA-based hydrothermal process emphasizes reductant-free chemical leaching and broad applicability across diverse cathode chemistries while maintaining ultrahigh extraction and recovery efficiency.

Considering the limitations associated with conventional organic acid leaching systems, including poor universality, low selectivity, and reliance on reducing agents, this study introduces a scalable and oxidant-free hydrothermal method for lithium recovery from a wide range of LIB cathode chemistries. Utilizing 1,2,4,5-benzenetetracarboxylic acid (BTCA) as the leaching agent, the proposed process enables complete lithium extraction from LFP within 1 h and achieves over 99% lithium recovery from NMC materials within 5 h. This approach is also applicable to other common cathode types, including lithium cobalt oxide (LiCoO_2_, LCO), lithium manganate (LiMn_2_O_4_, LMO), and mixed black mass (BM). The remaining solid residues are converted into reusable transition metal oxides, further enhancing resource utilization. In addition to its high leaching efficiency and material compatibility, the recyclability and reusability of the leaching agent, along with the simplified operation process, can reduce manufacturing costs and enhance economic feasibility. Collectively, these findings position the BTCA-based leaching strategy as a universal, efficient, and economically attractive solution for sustainable lithium recovery.

## 2 Experimental Section

### 2.1 Materials

The lithium extraction process employed a range of reagents and materials, including BTCA (Sigma) and commercial-grade lithium carbonate (Li_2_CO_3_, MTI), and various lithium-ion battery cathode compounds. The cathode materials comprised LiNi_1/3_Mn_1/3_Co_1/3_O_2_ (NMC111, MTI), LiNi_0.6_Mn_0.2_Co_0.2_O_2_ (NMC622, Umicore), LiNi_0.8_Mn_0.1_Co_0.1_O_2_ (NMC811, MTI), LiCoO_2_ (LCO, MTI), LiMn_2_O_4_ (LMO, MTI), and LiFePO_4_ (LFP, MTI). Additionally, the black mass was commercially sourced from a battery recycler in the United States of America, comprising a heterogeneous mixture of cathode materials, graphite, conductive carbon, and other components from end-of-life lithium-ion batteries. As the study did not involve dismantling of spent cells, dismantling-related pretreatment steps are not applicable. The detailed composition of all cathode materials is listed in Table S1. Zirconia (ZrO_2_, Sigma-Aldrich) and aluminum oxide (Al_2_O_3_, Sigma-Aldrich) were used as doping elements. Dimethyl sulfoxide (DMSO, Sigma-Aldrich, purity >99.7%) was employed as a selective solvent for recovering unreacted BTCA from the leachate. Acetone (Sigma-Aldrich, 99.5% purity) was used to facilitate the recrystallization of lithium products from the solution phase. Furthermore, battery-grade lithium carbonate (Sigma-Aldrich, >99.9%) served as a reference precursor for synthesizing a control sample of NMC622, enabling direct comparison with NMC622 produced from recovered lithium carbonate in this study.

### 2.2 Materials Characterization

Elemental concentrations in all leaching solutions, recycled chemicals, and final products were quantitatively analyzed using Inductively Coupled Plasma Optical Emission Spectrometry (ICP-OES) to assess purification and efficiency. The crystalline structures of the particles were examined through X-ray powder diffraction (XRD) using a PANalytical Empyrean system with a Cu Kα radiation source (λ = 1.54 Å) and a scanning increment of 0.0167°. Scanning Electron Microscopy (SEM; JSM 7000F SEM) was employed to analyze morphology and particle size. Nuclear Magnetic Resonance (NMR) spectroscopy was performed to confirm the chemical composition of the extracted product. The thermal behavior and compositional changes of Li_4_BTC contaminants were assessed using a Simultaneous Thermal Analyzer (TGA 1200), which records thermal transitions and mass variations with respect to temperature or time in a dry air environment. X-ray Photoelectron Spectroscopy (XPS) was conducted using a PHI 500 VersaProbe II apparatus from Physical Electronics to determine the oxidation states of metallic elements on particle surfaces, with spectral fittings processed via XPSpeak 41 software. Focused ion beam (FIB) imaging and advanced SEM were performed with a Thermo Scientific Scios 2 DualBeam system for cross-section preparation and high-resolution scanning.

### 2.3 Electrochemical Testing

The electrode slurry was prepared by dispersing active material (93 wt %), conductive carbon black (C65) at 4 wt %, and polyvinylidene fluoride (PVDF) binder constituting the remaining 3 wt % in *N*-methyl-2-pyrrolidone (NMP) solvent to form a uniform slurry. This slurry was then uniformly coated onto the aluminum foil and subsequently dried at 80 °C for 1 h in a conventional oven to remove solvent. After drying, the electrodes were calendared to achieve the desired density (2.7 g·cm^–3^) and then cut into square sheets of 57 mm by 44 mm for use in single-layer pouch cells. Each electrode was weighed with precision to ensure consistent mass loading, targeting approximately 18 mg·cm^–2^. Prior to cell assembly, the electrodes underwent further drying under vacuum at 120 °C for 12 h to eliminate moisture and residual solvent traces. Cell assembly was performed in an argon-filled glovebox to prevent effects from air or moisture. The pouch cell configuration included the prepared cathode, a graphite anode, a separator, and a suitable liquid electrolyte. The formation of cells was conducted at C/20, within a voltage window of 2.8–4.3 V. Electrochemical characterization of the assembled single-layer pouch cells was conducted across various current rates, ranging from C/20 to 2C, to evaluate rate capability within a voltage window of 2.8–4.2 V. Long-term cycling stability was tested at a constant current rate of 0.5C. All electrochemical measurements were carried out at ambient temperature.

### 2.4 Quality Control of Recovered Lithium Carbonate

To evaluate the manufacturability of the recovered lithium source, recycled NMC622 cathodes were synthesized using recovered battery-grade Li_2_CO_3_ obtained from this work (denoted as R-Li_2_CO_3_) and commercial battery-grade Li_2_CO_3_ purchased from Sigma as a reference (denoted as V-Li_2_CO_3_). In both cases, the same recycled NMC622 precursor was used to ensure a fair comparison.^45^ The cathode synthesized with recovered Li_2_CO_3_ is denoted as R-NMC622, while the control synthesized with commercial Li_2_CO_3_ is denoted as V-NMC622. For dopant/coating studies, 0.1 mol % ZrO_2_ was introduced during the lithiation step, and 0.35 wt % Al_2_O_3_ was applied as a postsinter coating. Briefly, Li_2_CO_3_ (R-Li_2_CO_3_ or V-Li_2_CO_3_) was mixed with the recycled NMC622 precursor to the targeted stoichiometry; for Zr-doped samples, 0.1 mol % ZrO_2_ was added during mixing. The mixtures were first calcined at 450 °C for 5 h, followed by sintering at 850 °C for 18 h in air. After sintering, the as-sintered powders were subsequently mixed with 0.35 wt % Al_2_O_3_ and recalcined at 450 °C for 5 h.

## 3 Results and Discussion

### 3.1 Establishment of the Recycling and Recovery Process

The BTCA-based lithium recycling process encompasses lithium extraction via hydrothermal reaction, followed by recovery and purification to obtain a battery-grade lithium source. Finally, the obtained recycled lithium source can be reused in battery manufacturing (as shown in Figure 1 and Figure S37). The lithium extraction process was conducted in a hydrothermal reactor designed to maintain high temperature and pressure conditions. Specifically, spent lithium-ion battery materials were mixed with water and BTCA in a polytetrafluoroethylene (PTFE) liner, which was then sealed within a stainless-steel autoclave. Under ambient conditions, BTCA exhibits poor solubility in both organic and inorganic solvents;^46^ however, its solubility can be significantly enhanced at elevated temperatures during hydrothermal treatment.^47^ Under hydrothermal conditions, the elevated temperature primarily enables BTCA to reach an effective dissolved concentration and to provide sufficient proton activity for delithiation reactions. Importantly, lithium selectivity in this system does not arise from solubility alone; rather, it is governed by coupled reaction and phase-partitioning behaviors: protons promote Li extraction from cathode lattices (proton-promoted delithiation), while transition-metal transfer to the liquid phase is strongly suppressed. To optimize the leaching process, experimental parameters, including temperature, solid-to-liquid (S/L) ratio, and BTCA concentration, were systematically varied. During the hydrothermal reaction, lithium ions are selectively extracted from layered (NMC) and olivine (LFP) cathode structures into the aqueous phase, forming soluble lithium salts. Meanwhile, transition metal oxides (such as transitional metal oxide and FePO_4_) and residual unreacted BTCA remain in the solid phase. After lithium extraction reaction, the slurry is filtered to separate a Li-enriched filtrate from the solid residue, which consists of residual transition-metal oxides mixed with unreacted BTCA. Lithium salts in the Li-enriched filtrate are then recovered by acetone-induced recrystallization as either tetralithium 1,2,4,5-benzenetetracarboxylate (Li_4_BTC) or Li_3_PO_4_, depending on the anion composition. Finally, the sintering of Li_4_BTC powder under an air atmosphere was carried out according to the reaction eq 1, during which Li_4_BTC reacts with oxygen to form Li_2_CO_3_ and release CO_2_.^48^ The transition metal 1,2,4,5-benzenetetracarboxylate also reacts similarly with oxygen to form metal oxides and CO_2_ (eq 2)^46,48^

1 $${\mathrm{Li}}_{4}C_{10}H_{2}O_{8}+\frac{15}{2}O_{2}\to 2{\mathrm{Li}}_{2}CO_{3}+8CO_{2}↑+H_{2}O$$

2 $$TM_{x}C_{10}H_{2}O_{8}+7O_{2}\to TM_{x}O+10CO_{2}↑+H_{2}O$$

**Figure 1:**
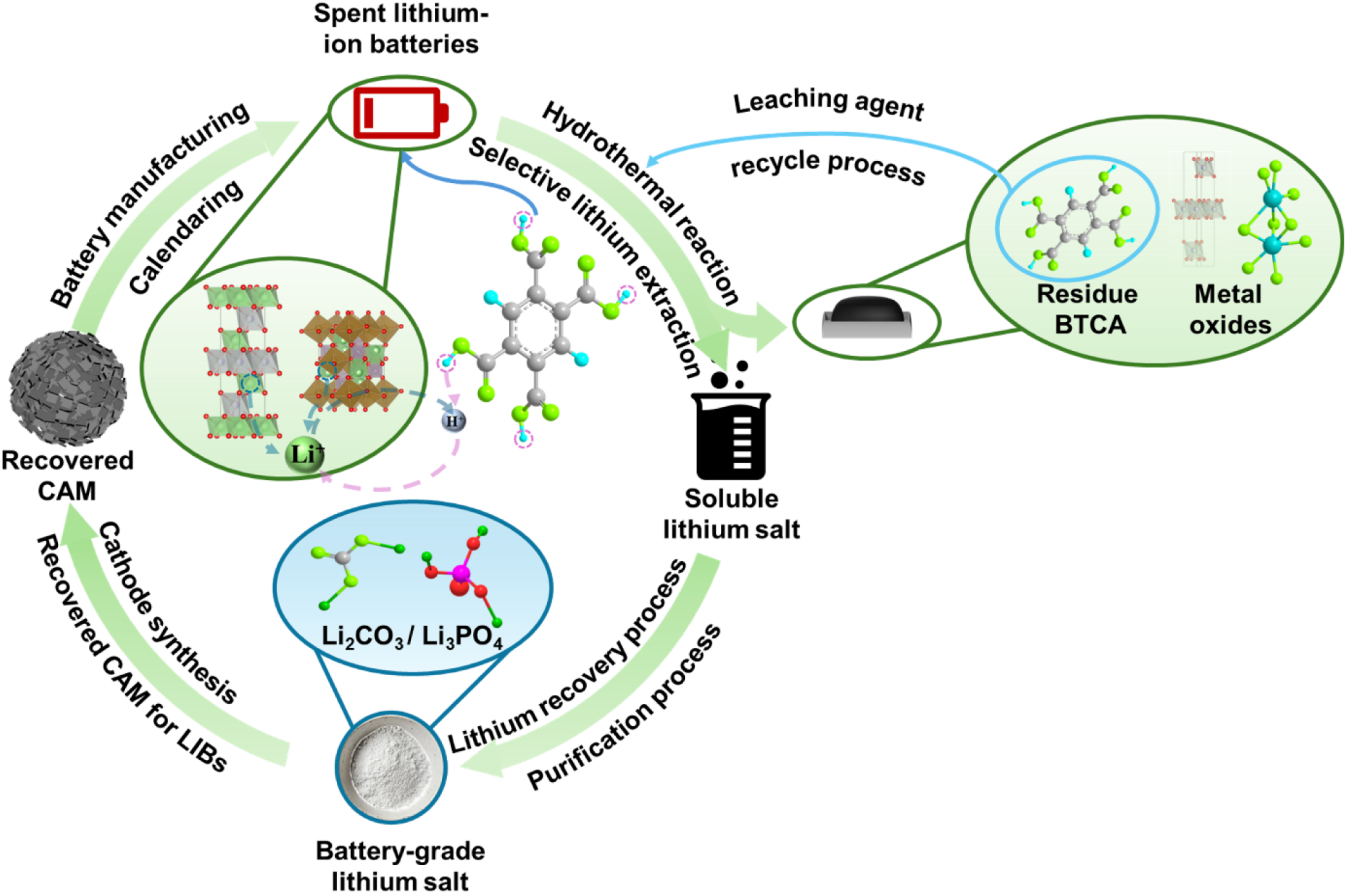
Recycling and recovery process for BTCA based lithium extraction reaction.

The thermal treatment was conducted by gradually raising the temperature at a rate of 5 °C per minute until reaching 700 °C. After calcination, the calcined powder consisted primarily of Li_2_CO_3_ along with trace insoluble transition-metal oxides originating from minor coextracted TM species. The powder was dispersed in deionized water at ambient conditions, and a dissolution–filtration step was performed to dissolve Li_2_CO_3_ into the aqueous phase while removing insoluble oxide residues, thereby improving product purity. This solution was then introduced into acetone to induce crystallization of lithium carbonate through antisolvent precipitation. The precipitated lithium carbonate was separated via filtration, rinsed thoroughly with acetone to remove impurities, and subsequently dried in a conventional oven. To enable reuse of the organic solvent, the spent acetone was purified by distillation, taking advantage of the distinct boiling points of its components.^45^

The recovered lithium carbonate was further synthesized with a recycled precursor, which was prepared based on our previous work. For comparison, virgin lithium carbonate was also included. The lithium carbonate was first mixed with the NMC622 precursor and 0.1 mol % ZrO_2_ dopant, then sintered at 450 °C for 5 h, followed by a second sintering at 850 °C for 18 h. The resulting cathode materials were subsequently mixed with 0.35 wt % Al_2_O_3_ as a coating material and resintered at 450 °C for 5 h.^49^ Lastly, to enable recovery and reuse of the organic chelating agent, the solid byproduct, comprising residual BTCA and transition metal oxides, was subjected to dissolution in dimethyl sulfoxide (DMSO), a solvent in which BTCA is highly soluble.^48^ The solution was then filtered to separate undissolved solids, and BTCA was subsequently recovered by introducing water, a nonsolvent that induced its recrystallization. This process yielded high-purity BTCA suitable for reuse in further extraction cycles. The remaining inorganic residue primarily consisted of metal oxides and could be directed toward additional purification steps or repurposed directly for cathode material synthesis. Overall, this integrated approach supports a closed-loop recovery system, achieving selective lithium separation, organic ligand regeneration, and transition metal valorization, thereby promoting environmentally conscious and resource-efficient battery recycling.

### 3.2 Novel Lithium Extraction and Recovery for LFP

To achieve high lithium leaching efficiency while maintaining excellent selectivity, a series of leaching parameters was systematically optimized, including the acid concentration, reaction temperature, and solid-to-liquid (S/L) ratio. As shown in Figure S1, the pristine LFP consists of nanoscale particles with a uniform distribution of Fe and P elements, and a thin carbon coating is clearly observed on the particle surfaces. Initially, the reaction parameters were set to a reaction time of 1 h, a temperature of 210 °C, and an S/L ratio of 1:20.

The leaching performance was strongly influenced by the concentration of BTCA, as illustrated in Figure 2a. Increasing the acid concentration significantly enhanced the dissolution of both lithium and phosphorus. At BTCA concentrations below 0.2 M, the leaching efficiencies of Li and P remained relatively low and nearly equivalent, as shown in Figures 2b, S2, and S3. XRD analysis of the solid residues confirmed that the LFP crystal structure was largely preserved under these low-acid conditions. As the BTCA concentration increased above 0.2 M, new crystalline phases such as FeHPO_4_ and Fe_2_O_3_ began to appear in the XRD patterns, indicating partial decomposition of LFP.^50^ Elemental mapping revealed a decrease in phosphorus content within the residue, while iron remained more concentrated, suggesting preferential leaching of phosphorus and incomplete conversion of Fe-containing intermediates at intermediate acid levels.^51^ At a BTCA concentration of 0.45 M, the XRD results revealed complete transformation of the solid phase to Fe_2_O_3_, with no residual LFP peaks detected. Under these conditions, leaching efficiencies reached 100% for lithium and 96.06% for phosphorus, indicating near-complete extraction of lithium with minimal coleaching of iron.

**Figure 2:**
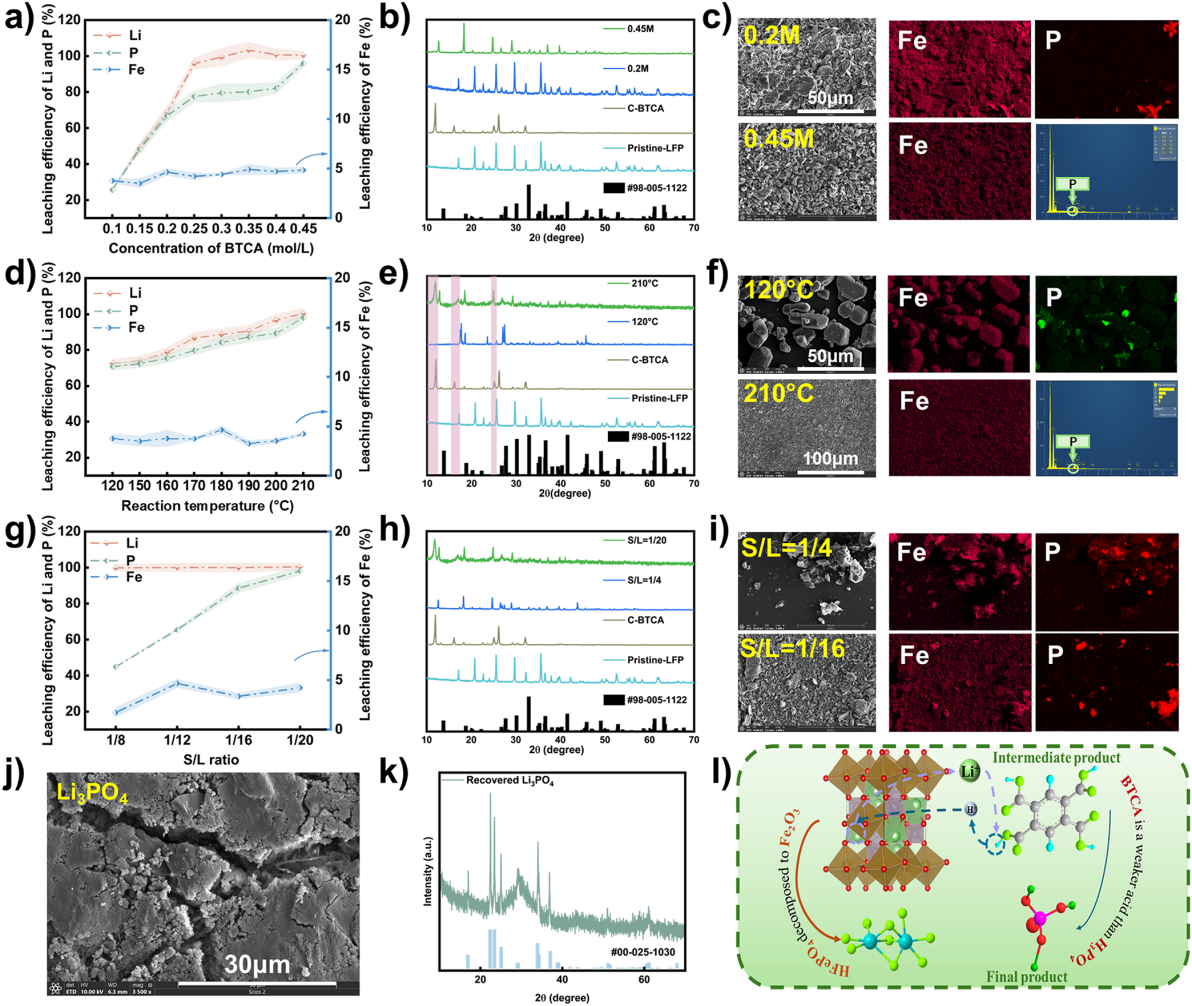
a) Extraction of Li, P and Fe under different concentration of BTCA, b) XRD pattern for residue powder under different concentration of BTCA, c) SEM images for residue powder under different concentration of BTCA, d) extraction of Li, P and Fe under different reaction temperature, e) XRD pattern for residue powder under different reaction temperature, f) SEM images for residue powder under different reaction temperature, g) extraction of Li, P and Fe under different S/L ratio, h) XRD pattern for residue powder under different S/L ratio, i) SEM images for residue powder under different S/L ratio, j) SEM images for lithium product, k) XRD pattern for lithium product, and l) the mechanism of lithium extraction reaction from LFP.

To further improve energy efficiency, the reaction temperature was systematically optimized. As shown in Figures 2c, 2d, S4, and S5, microscale cubic particles identified as FeHPO_4_ via XRD were generated when the temperature was below 180 °C, and unreacted LFP particles were still visible on their surfaces, indicating incomplete decomposition. To further corroborate the formation of FeHPO_4_, NMR analysis (Figure S6) was conducted on the residue powder obtained at 150 °C. The residue powder was dissolved in deuterated sulfuric acid diluted with deuterium oxide (D_2_O), while BTCA dissolved in the same solvent served as the pristine reference. As BTCA is insoluble in dilute sulfuric acid,^52^ the filtrate obtained after dissolution was used for testing. In the ^1^H NMR spectrum of the 150 °C product, an additional proton signal was observed compared with the pristine sample, and a phosphorus peak was detected in the ^31^P NMR spectrum. Combined with the XRD test results, these NMR findings further confirm the formation of FeHPO_4_ at 150 °C. At temperatures above 180 °C, flake-like particles began to form, which were confirmed as Fe_2_O_3_ by XRD analysis. At 210 °C, the phosphorus signal was no longer detected by EDS mapping, and the XRD patterns displayed only Fe_2_O_3_ peaks, confirming full decomposition of FeHPO_4_ and complete release of phosphorus into the leachate.^50^ Therefore, 210 °C was selected as the optimal temperature, ensuring full conversion of LFP while avoiding unnecessary energy consumption.

Finally, the S/L ratio was optimized to reduce reagent consumption and enhance processing capacity (Figures 2e, 2f, S7, and S8). At higher S/L ratios (i.e., lower liquid volumes), the decomposition of FeHPO_4_ was incomplete, as evidenced by persistent FeHPO_4_ peaks in the XRD patterns and uniform phosphorus distribution in SEM-EDS mapping. These results indicate delayed FeHPO_4_ breakdown under limited solution volume, which negatively impacts phosphorus recovery. To ensure complete extraction and high selectivity, the S/L ratio was optimized and fixed at 1:20.

Based on the optimization results, the final reaction conditions were determined as follows: temperature of 210 °C, time of 1 h, BTCA concentration of 0.45 M, and S/L ratio of 1:20. Due to the large amount of BTCA remaining in the residues, the XRD pattern was dominated by the diffraction peaks of BTCA, making other characteristic peaks less discernible. To obtain a clearer XRD characterization of the Fe-containing product, the residues obtained under the optimal conditions were first dissolved in DMSO to remove the residual BTCA, and then the resulting powder was heated at 120 °C under an Ar atmosphere to evaporate any remaining DMSO while avoiding oxidation of the Fe product. As shown in Figure 2g, distinct diffraction peaks corresponding to Fe_2_O_3_ were clearly observed, which are in excellent agreement with the standard reference pattern (PDF card No. 98-005-6123). Under these conditions, the leaching efficiency reached 100% for lithium and 96.32% for phosphorus, with only 4.32% of iron coextracted, indicating high selectivity. After filtration, the resulting leachate containing dissolved lithium and phosphorus was collected for further processing.

To promote the decomposition of residual FeHPO_4_ in the leachate, the solution was transferred into a PTFE-lined autoclave and subjected to additional hydrothermal treatment. As illustrated in Figure S9, increasing the reaction temperature led to greater conversion of FeHPO_4_ into FePO_4_ due to the lower H^+^ concentration in the solution,^50^ thereby improving separation and purification outcomes. Subsequently, the processed leachate was poured into acetone to induce precipitation of the lithium salt through an antisolvent recrystallization approach. As shown in Figure 2h, the recrystallized lithium salt formed uniform nanoscale particles. XRD analysis confirmed that the crystalline product was pure lithium phosphate (Li_3_PO_4_), with no detectable impurity phases. However, due to the nanoscale particle size of the Li_3_PO_4_, the diffraction peak around 30° in the XRD pattern appeared broadened. The chemical purity of the recovered Li_3_PO_4_ was further evaluated using ICP-OES (listed in Table S2). Comparable iron contamination with commercial Li_3_PO_4_ (C-Li_3_PO_4_) was detected, and the lithium phosphate purity reached 99.95%, meeting the specifications for battery-grade materials. Although Li_3_PO_4_ is not a direct lithium source for NMC cathode synthesis, it can serve as a practical lithium intermediate. Previous studies have shown that Li_3_PO_4_ can be converted into conventional lithium salts such as Li_2_CO_3_ or LiOH.^53,54^ In addition, Li_3_PO_4_ has also been reported to be directly used as the lithium source for LFP resynthesis.^55^

Therefore, the proposed mechanism of lithium extraction is illustrated in Figure 2i. At elevated temperatures, BTCA dissolves in water and dissociates to release protons (H^+^) into the solution. These protons subsequently undergo the proton-promoted delithiation process, where lithium release can additionally involve acid-promoted decomposition and intermediate phase evolution, which is also supported by the experimentally observed differences in lithium-product identity and residue-phase evolution. Under the acidic, H^+^ rich conditions, the intermediate FeHPO_4_ becomes unstable and is further decomposed into Fe_2_O_3_. Concurrently, phosphate ions (PO_4_^3–^) generated during the reaction combine with the extracted lithium ions to form Li_3_PO_4_.

### 3.3 Novel Lithium Extraction and Recovery for NMC

For LFP, phosphate species released during hydrothermal delithiation drive the extracted Li^+^ to precipitate as Li_3_PO_4_ during antisolvent recrystallization; in contrast, phosphate-free NMC leachates yield Li_4_BTC via association of Li^+^ with BTC^4–^. The extraction reaction for NMC materials was conducted under initial conditions of 210 °C, 5 h of reaction time, an S/L ratio of 1:20, and a 100% excess of BTCA, calculated based on the molar ratio of H^+^ to lithium ions (Li^+^). The detailed stoichiometric calculations are provided in Table S3. As shown in Figures 3a–c, S10, and S11, increasing the reaction temperature resulted in enhanced lithium extraction efficiency, with efficiency reaching up to 99.15%. In contrast, the leaching efficiencies of transition metals (total amount of Ni, Mn, and Co) decreased at higher temperatures, indicating improved selectivity. XRD analysis of the solid residue revealed that the primary diffraction peaks aligned well with the pristine NMC622 phase, while an additional peak near ∼17° indicated the presence of transition metal oxides.^47^ Because the postleaching solid residue contains both extracted cathode solids and residual BTCA, unambiguous direct spectroscopic identification of transition metal-BTCA complexes (TM (Ni, Mn, and Co)-1,2,4,5-benzenetetracarboxylate (TM_*x*_BTC)) from the residue is experimentally challenging. Without washing, BTCA functional-group signals would dominate any vibrational spectra and are not uniquely diagnostic of metal-carboxylate coordination. However, aggressive washing to remove BTCA can simultaneously remove weakly associated TM_*x*_BTC intermediates, thereby eliminating the species of interest. Therefore, we interpret TM_*x*_BTC intermediate formation based on consistent indirect evidence, including ligand identity in solution and chemical-state evolution in the leached solids, together with the observed suppression of TM coleaching. Bulk XPS measurements (Figure S34) further show a pronounced increase in the Ni^2+^ fraction in the leached solids relative to the pristine bulk, consistent with the surface-sensitive XPS trend and supporting the proposed presence of a divalent-Ni-enriched chemical environment after leaching. Furthermore, NMR analysis (Figure S18) detects only BTC-related functional groups, indicating that BTC^4–^ is the only organic anionic ligand present in the leachate; thus, any dissolved transition-metal species must be associated with BTC^4–^ rather than alternative ligands. SEM images further confirmed that elevated temperatures induced crack formation on the surface of NMC particles, which likely facilitated proton diffusion and promoted lithium extraction via enhanced proton-promoted delithiation pathways. The amount of excess BTCA was also optimized to minimize chemical consumption while maintaining high efficiency. As shown in Figures 3d–f, S12, and S13, increasing BTCA content improved both lithium and transition metal leaching efficiencies, indicating enhanced formation of TM_*x*_BTC complexes. Notably, at 10% excess BTCA, lithium leaching reached its maximum efficiency, while coextraction of transition metals was minimized. Therefore, 10% excess BTCA was identified as the optimal condition to balance efficiency and selectivity. The reaction time was further optimized to reduce energy usage while preserving performance. As presented in Figures 3g–i, S14, and S15, shorter reaction times resulted in decreased lithium recovery and higher transition metal leaching. This was attributed to incomplete decomposition of TM_*x*_BTC complexes.^47^ Therefore, a reaction time of 5 h was selected to ensure complete lithium extraction with minimal coextraction of transition metal. Under these optimized conditions, lithium leaching efficiency reached 99.15%, with only 0.04% of transition metals coextracted.

**Figure 3:**
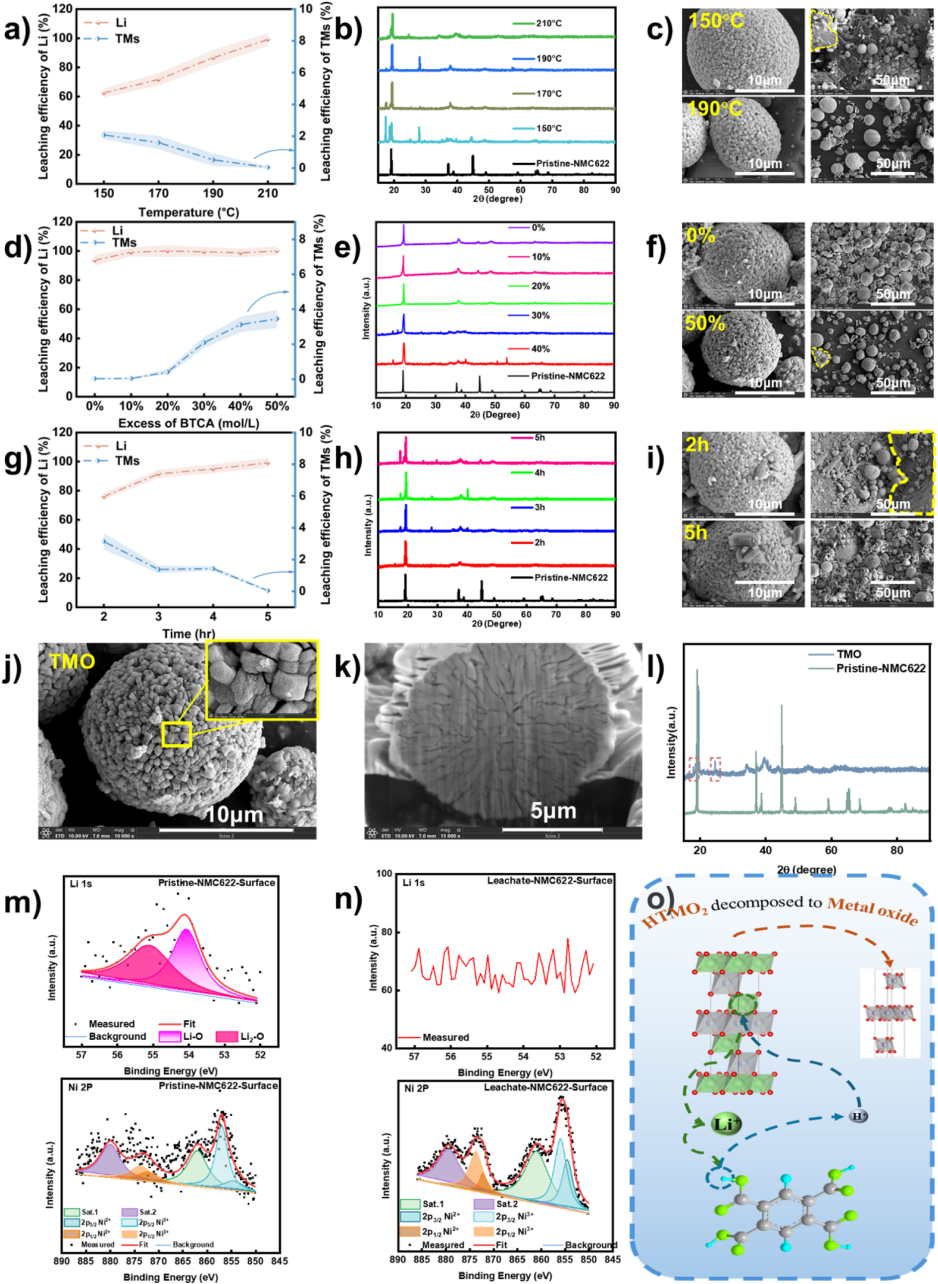
a) Extraction of Li and transition metal under different temperature, b) XRD pattern for residue powder under different temperature, c) SEM images for residue powder under different temperature, d) extraction Li and transition metal under different excess of BTCA, e) XRD pattern for residue powder under different excess of BTCA, f) SEM images for residue powder under different excess of BTCA, g) extraction of Li and transition metal under different time, h) XRD pattern for residue powder under different time, i) SEM images for residue powder under different time, j) SEM images transition metal oxide, k) cross section SEM images of transition metal oxide, l), XRD pattern comparison of transition metal oxide and pristine NMC622, m) XPS spectrum of Li 1s and Ni 2p for pristine NMC622, n) XPS spectrum of Li 1s and Ni 2p for transition metal oxide, and o) the mechanism of lithium extraction reaction from NMC.

Figures 3j and 3k highlight the formation of extensive surface and internal cracks in the postextraction transition metal oxide (TMO), as observed in SEM and cross-sectional images. These structural features increase the diffusion pathways, further enhancing lithium extraction. XRD analysis (Figure 3l) showed additional peaks, which are highlighted in the red square, corresponding to transition metal oxides. Rietveld refinement results (Figure S16 and Table S4) confirmed that the TMO retained a layered structure with an expanded *c*-axis lattice parameter, consistent with full lithium deintercalation and lattice expansion.^56^ XPS further validated the extraction results. As shown in Figures 3m and 3n, the Li 1s spectrum of the pristine material displayed two distinct peaks corresponding to residue lithium on the surface of the cathode (Li–O) and lithium in the lattice (Li_2_–O) species.^2^ In contrast, no lithium peaks were detected in the TMO, indicating nearly complete lithium removal. XPS spectra of the transition metals (Figure 3n and Figure S17) revealed an increased proportion of lower oxidation states (Ni^2+^, Mn^2+^, and Co^2+^), consistent with the formation of transition metal oxides during the decomposition of the corresponding TM_*x*_BTC complexes.

The proposed mechanism of the extraction process is depicted in Figure 3o. At elevated temperatures, BTCA dissolves in water, releasing H^+^ ions that exchange with Li^+^ in the NMC structure. Simultaneously, TM_*x*_BTC coordination complexes form but are thermally unstable and decompose into transition metal oxides, resulting in a reduction in the average oxidation states of Ni, Mn, and Co. NMR analysis (Figure S18) confirmed that the lithium-containing product in the leachate was Li_4_BTC, supporting the proposed reaction pathway.

### 3.4 Universal Usage and Application of Extraction Products

The broad applicability of the BTCA-based extraction method was further validated using lithium manganese oxide (LiMn_2_O_4_, LMO), lithium cobalt oxide (LiCoO_2_, LCO), and black mass, a cathode mixture primarily composed of NMC111 and LMO, sourced from a commercial battery recycler. As shown in Figure S19, under optimized leaching conditions, the lithium leaching efficiencies reached 98.50% for LMO, 98.95% for LCO, and 94.06% for black mass. Simultaneously, the coleaching of transition metals was effectively suppressed, with extraction efficiencies of only 0.23%, 0.53%, and 0.72% for LMO, LCO, and black mass, respectively, highlighting the high selectivity and universal usage of the BTCA system.

SEM imaging revealed notable morphological transformations in the solid residues compared to the pristine feedstocks (Figure 4a), along with the presence of residual BTCA on the particle surfaces (Figure S20). Complementary XRD analysis (Figure 4b) confirmed the identity of the postleaching products. The extracted LiCoO_2_ (LCO) still exhibits characteristic diffraction peaks corresponding to the layered LCO structure. However, the significant weakening of the (003) reflection indicates a high degree of delithiation.^57^ In addition, the presence of diffraction peaks corresponding to Co_3_O_4_ (PDF card #98-005-6123) confirms the decomposition of Co_*x*_BTC complexes during processing. In contrast, the original spinel structure of LiMn_2_O_4_ (LMO) is no longer detectable in the extracted LMO powder. Instead, new reflections corresponding to a tetragonal phase (PDF card #98-008-7774) are observed, suggesting a structural transformation of the LMO component.^58^ Furthermore, peaks assigned to MnO (PDF card #98-006-1319) are evident, indicating further decomposition of the Mn_*x*_BTC complexes and possible reduction of manganese species. The analyzed black mass was composed of a mixture of NMC111 and LMO cathode materials. Therefore, the XRD pattern reveals the presence of a degraded layered structure (from NMC), tetragonal phase LMO, and various transition metal oxides, all indicative of structural degradation and transformation during the extraction and recovery process. These results confirm that the BTCA-mediated process is not only highly effective for lithium recovery but also adaptable to various cathode chemistries and complex industrial mixtures.

**Figure 4:**
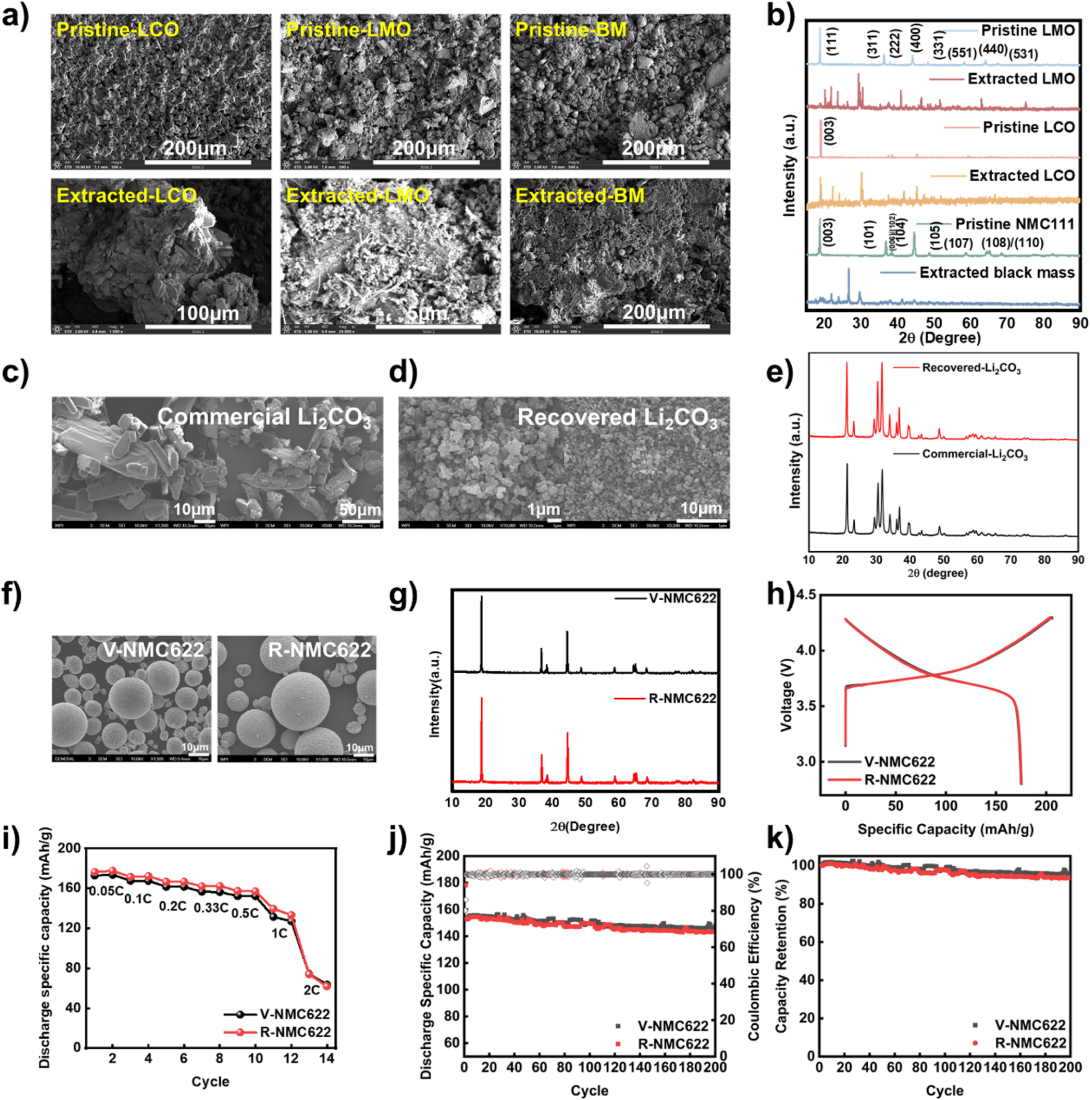
a) SEM images of pristine and extracted LMO, LCO, and black mass, b) XRD pattern for pristine and extracted LMO, LCO, and black mass, c) SEM images for commercial lithium carbonate, d) SEM images for recovered lithium carbonate, e) XRD pattern for comparison of recovered lithium carbonate and commercial lithium carbonate, f) SEM images for V-NMC622 cathode powder and R-NMC622 cathode powder, g) XRD pattern comparison for R-NMC622 powder and V-NMC622 powder, h) formation comparison for R-NMC622 powder and V-NMC622 powder, i) rate performance comparison for R-NMC622 powder and V-NMC622 powder, j) cyclic performance comparison for R-NMC622 powder and V-NMC622 powder, and k) discharge specific capacity retention comparison for V-NMC622 and R-NMC622 powder.

The Li_4_BTC powder was subsequently converted into lithium carbonate (Li_2_CO_3_) through a sintering-recrystallization route. During sintering, Li_4_BTC decomposed into lithium carbonate along with manganese, cobalt, and nickel oxides. TGA (Figure S21) confirmed the decomposition profile, showing no mass loss below 100 °C, thereby indicating the absence of crystalline water in the recrystallized crude Li_4_BTC. A distinct weight loss near 300 °C was observed, corresponding to the thermal decomposition of the organic ligand. The total weight loss was approximately 38%, which is in close agreement with the theoretical value calculated for the conversion of Li_4_BTC to Li_2_CO_3_.^48^

After calcination of the crude recrystallized Li_4_BTC, the resulting solid was dispersed in DI water to selectively dissolve Li_2_CO_3_, leaving transition metal oxides as insoluble residues. Filtration separated the solid oxides from the lithium-rich solution. To isolate the lithium carbonate, the filtrate was mixed with acetone (volume ratio for water:acetone was 1:1), leading to precipitation of Li_2_CO_3_ due to its low solubility in organic solvents. The precipitate was collected via filtration to obtain pure lithium carbonate. SEM revealed that the recovered lithium carbonate exhibited smaller and more uniform particle sizes compared to the commercial Li_2_CO_3_, potentially enhancing its dispersion in precursor formulations (Figures 4c and 4d).^45^ XRD analysis confirmed that the crystalline phase of the recovered Li_2_CO_3_ was consistent with that of the commercial reference (Figure 4e). To assess chemical purity, ICP-OES analyses were performed on equal masses of commercial and recovered lithium carbonate dissolved in aqua regia. As summarized in Table S5, the recovered lithium carbonate exhibited significantly lower impurity levels. The final purity was determined to be 99.97%, slightly surpassing the commercial grade, which was measured at 99.92%.

As defined in Section 2.4, R-NMC622 denotes cathodes synthesized with recovered Li_2_CO_3_, while V-NMC622 denotes cathodes synthesized with commercial Li_2_CO_3_ using the same recycled NMC622 precursor. To assess the applicability of the recovered battery-grade lithium carbonate, a batch of LiNi_0.6_Mn_0.2_Co_0.2_O_2_ cathode material (R-NMC622) was synthesized using the reclaimed lithium carbonate in combination with a recycled NMC622 precursor. For comparison, a reference sample (V-NMC622) was synthesized using commercial lithium carbonate and the same recycled precursor. Both cathode materials were subjected to identical synthesis conditions: initial calcination at 450 °C for 5 h, followed by sintering at 850 °C for 18 h. After sintering, the powders were dried under vacuum at 120 °C for 12 h to eliminate residual moisture and oxygen. Electrodes were fabricated using both R-NMC622 and V-NMC622 to evaluate their electrochemical properties. SEM imaging (Figure 4f) revealed that both materials exhibited comparable morphologies, consisting of spherical secondary particles composed of well-defined primary crystallites. No significant morphological differences were observed between the recovered and reference samples. XRD analysis (Figure 4g) confirmed the high crystallinity and phase purity of both samples, with no detectable impurity peaks. Both R-NMC622 and V-NMC622 retained the characteristic α-NaFeO_2_-type layered structure. The presence of clearly resolved (006)/(102) and (108)/(110) peak splitting further confirmed the well-ordered layered architecture in both samples. Rietveld refinement results (Figure S22, Table S6) showed that the lattice parameters of R-NMC622 closely matched those of the V-NMC622, indicating structural consistency. To substantiate the ZrO_2_-doping and Al_2_O_3_-coating strategies, additional characterization was performed for the modified NMC622 samples. SEM-EDS mapping confirmed the presence and uniform distribution of Zr and Al relative to both cathodes. The measured ICP-OES are consistent with the targeted dopant/coating loadings (Figures S35 and S36, Table S8), collectively supporting successful modification.

Electrochemical tests were carried out using single-layer pouch full cells, each with a cathode mass loading of 18 mg·cm^–2^. Initial charge–discharge measurements (Figure 4h) demonstrated that R-NMC622 delivered an initial discharge capacity of 175.3 mA h·g^–1^ and a Coulombic efficiency of 85.97%, which were nearly identical to those of V-NMC622 (174.9 mA h·g^–1^ and 84.99%, respectively). In the rate capability evaluation (Figure 4i), R-NMC622 showed discharge capacities of 177.4, 173.4, 168.1, 164.4, 160.2, 149.1, and 75.3 mA h·g^–1^ at current rates of 0.05C, 0.1C, 0.2C, 0.33C, 0.5C, 1C, and 2C, respectively. These values were closely aligned with those of V-NMC622, which delivered 175.1, 170.8, 165.8, 162.0, 157.8, 143.8, and 58.7 mAh·g^–1^ at the corresponding rates. Cycling stability tests (Figures 4j and 4k), conducted at a rate of 0.5C, demonstrated excellent performance for both materials. After prolonged cycling, R-NMC622 exhibited a capacity retention of 96.53%, slightly outperforming V-NMC622, which retained 96.66% of its initial capacity.

### 3.5 Carbon Footprint and Cost Analysis

A comprehensive carbon footprint and cost analysis was conducted to assess the economic and environmental viability of the BTCA-based lithium recovery process. Given that chemicals are the primary contributors to both energy consumption and greenhouse gas (GHG) emissions, the analysis focused predominantly on chemical inputs. To reduce reagent usage, the process incorporates the recovery and reuse of unreacted BTCA. After lithium extraction, BTCA and byproducts remain in the filtrate. By leveraging differences in solubility, dimethyl sulfoxide (DMSO) was employed to selectively dissolve residual BTCA. Subsequent recrystallization in water, where BTCA has low solubility, enabled its recovery in high purity. As shown in Figure S23, the recovered BTCA particles were smaller than their commercial counterparts, while EDS mapping detected no impurities. Further validation by ICP-OES (Table S7) also confirmed the absence of metallic contaminants. Structural analyses via XRD and NMR (Figures S24 and S25) revealed no changes in the crystal structure or functional groups, indicating that the recovered BTCA retained properties identical to the commercial standard. These findings demonstrate the chemical stability, reusability, and cost-effectiveness of the BTCA reagent, further strengthening the sustainability credentials of the process.

Cost analysis was performed using the EverBatt model developed by Argonne National Laboratory, integrating the most recent and representative data sets. The model compared the BTCA-based recycling approach with conventional hydrometallurgical processes. The analysis encompassed the entire battery recycling workflow, including lithium recovery, precursor synthesis, and cathode material production. Figures S26 and S27 detail the BTCA-lithium recovery pathway and contrast it with the conventional method (Figures S28 and S29), which involves sodium carbonate precipitation and carbon dioxide purification to produce battery-grade lithium carbonate.^59^ Identical synthesis steps for precursors and cathodes were adopted for both methods, following protocols reported in prior studies.^60^ As illustrated in Figure 5a, the BTCA-based method demonstrated significantly lower costs and higher revenues compared to the traditional route. For a processing scale of 30,000 t per year of NMC622 black mass, the BTCA process incurred a cost of $1.16 per kilogram of feedstock, less than half the $2.72/kg cost of the conventional approach. Additionally, the BTCA route yielded higher revenues at $1.65/kg versus $1.485/kg for the traditional method, owing to superior lithium recovery efficiency. Although lithium is not the most valuable component in spent batteries, its recovery via BTCA contributed to a 40.4% increase in total revenue. For LFP feedstock, the cost of the BTCA method was also significantly lower ($0.86/kg) compared to $2.80/kg for the conventional process. Figure 5b compares the profitability of BTCA and traditional methods across different feedstock types. Under the BTCA-based process, total profit for the NMC622 black mass was $1.23/kg, the highest among all scenarios. Specific profits were $0.49/kg for NMC and $0.25/kg for LFP feedstocks, confirming the broad applicability of the method across chemistries. In contrast, the traditional process yielded a total profit of $0.69/kg only under overall recycling and recovery scenarios. Furthermore, the cost of incurred losses of $1.24/kg for NMC and $1.80/kg for LFP underscores the limited economic competitiveness of current recovery technologies.

**Figure 5:**
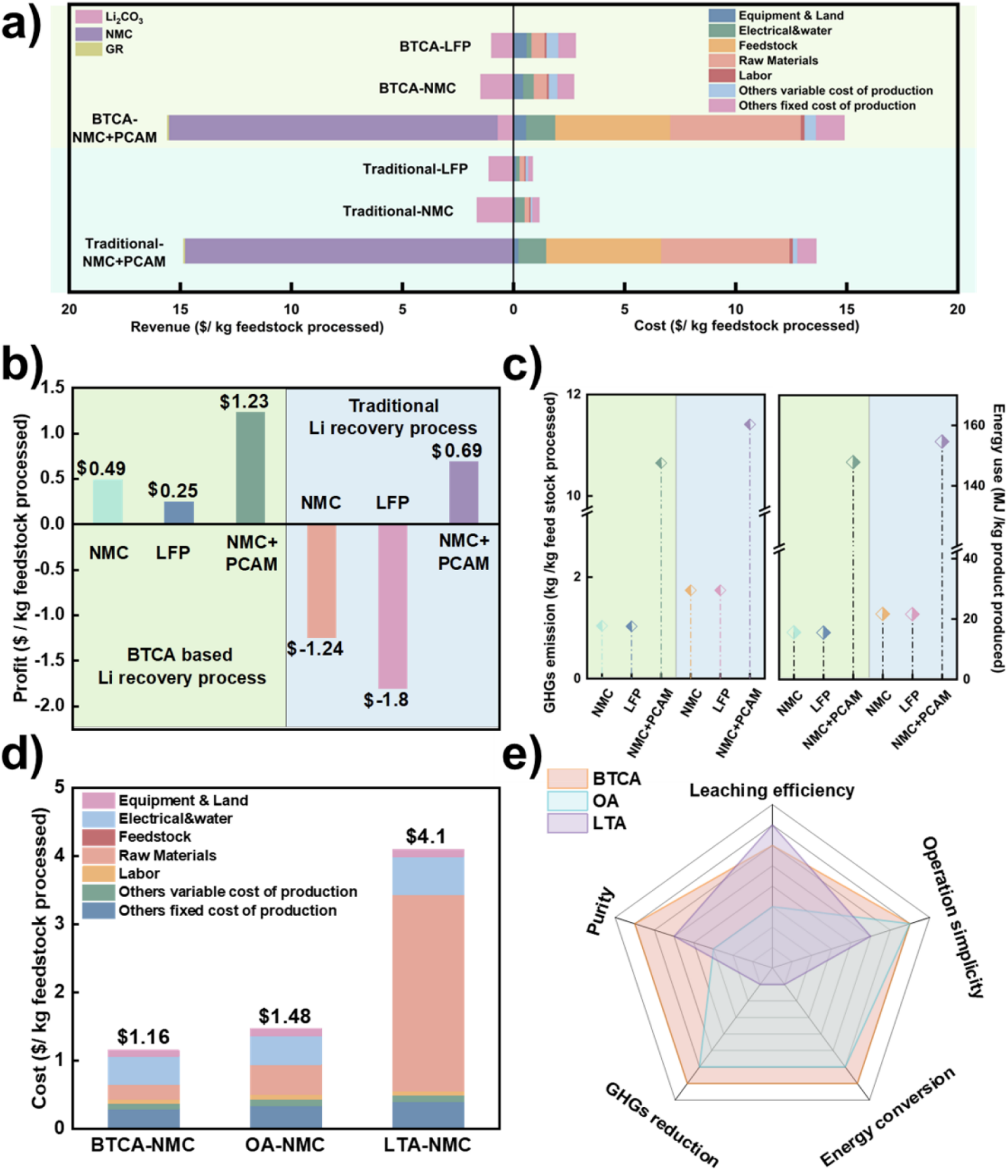
Cost analysis for different recycle and recover processes: a) total cost and total revenue comparison for BTCA-lithium recovery process with CAM synthesizing process and traditional-lithium recovery process with CAM synthesizing process; b) profit comparison for BTCA-lithium recovery process and traditional-lithium recovery process; c) global warming potential comparison for cathode synthesizing process, including greenhouse gas emission (GHGs) and energy consumption for traditional-lithium recovery process, and BTCA-lithium recovery process; d) total cost for BTCA-NMC recycling, OA-NMC recycling, and LTA-NMC recycling process; e) comprehensive comparison of different organic acid recycling technologies.

Environmental impact metrics, shown in Figure 5c, further highlight the advantages of the BTCA method. For both NMC and LFP feedstocks, GHG emissions were reduced by approximately 38.8% relative to the conventional method, while the overall recycling process, including lithium and cathode recovery, saw a net reduction of 6.8%. In terms of energy consumption, the BTCA-based process achieved a 28.26% reduction for NMC and LFP and a 4.4% reduction for the full recycling route compared to the baseline.

To further evaluate the economic feasibility and address potential concerns regarding the cost-effectiveness of BTCA compared with other reported organic acids, a detailed techno-economic analysis was performed using the EverBatt model (Figures 5d, 5e, and S30–S33). The BTCA-based recycling route for NMC exhibits a total processing cost of $1.16 kg^–1^ feedstock, which is lower than that of oxalic acid (OA, $1.48 kg^–1^) and L-tartaric acid (LTA, $4.10 kg^–1^) under an annual throughput of 30,000 t.^61,62^ As shown in Figure 5e, the BTCA system achieves a balanced performance among leaching efficiency, product purity, energy conversion, operation simplicity, and GHG reduction. Although BTCA is a specialty chemical, its superior selectivity, high-purity product yield, and mild reaction conditions offset its higher reagent cost, leading to an overall cost advantage. In contrast, OA offers lower reagent cost but limited leaching efficiency and poorer product purity, while LTA provides higher leaching efficiency but involves complex processing, high energy consumption, and elevated GHG emissions. Overall, the BTCA-based hydrothermal leaching process presents a well-balanced and cost-effective advancement over conventional organic acid–based lithium recovery methods.

## 4 Conclusion

In this work, we develop a universal and closed-loop hydrothermal recycling strategy using BTCA for the highly selective and efficient extraction of lithium from diverse cathode chemistries, including LFP, NMC, LMO, LCO, and industrial black mass. The BTCA-mediated process achieves near-complete lithium leaching (up to 100% for LFP and 99.15% for NMC) with minimal coextraction of transition metals (<1%), enabled by a proton-promoted delithiation mechanism under hydrothermal conditions. Following extraction, lithium is recovered as high-purity Li_3_PO_4_ or Li_4_BTC and subsequently converted to battery-grade Li_2_CO_3_ with 99.97% purity. Metal oxides are concurrently formed and recovered as valuable byproducts. The process is fully regenerative, with BTCA successfully recovered via DMSO-assisted crystallization without structural degradation. Furthermore, the recycled lithium carbonate was used to synthesize NMC622 cathode materials that demonstrated electrochemical performance comparable to those made with commercial lithium carbonate. Economic and environmental evaluations via the EverBatt model revealed that the BTCA-based process significantly reduces recycling costs and greenhouse gas emissions compared to conventional hydrometallurgical methods, with up to 40.4% higher revenue and 38.8% lower GHG emissions. This work presents a scalable and sustainable recycling pathway that addresses the increasing demand for lithium-ion battery material recovery, reduces reliance on primary lithium sources, and mitigates geopolitical risks associated with critical raw material supply chains.

## Supplementary Material



## Data Availability

The data supporting the findings of this study were generated from experiments conducted by the authors. All relevant data sets have been included in the manuscript and Supporting Information. Additional raw data and analysis scripts are available upon reasonable request from the corresponding author.

## Notes

Supplementary morphology, elemental mapping, and structural characterization of extracted LFP and NMC under different reaction conditions; NMR evidence of intermediate and lithium-containing products; XPS analysis of extracted NMC; leaching efficiencies for LCO, LMO, and real black mass; characterization of pristine and extracted feedstocks under optimized conditions; TGA analysis of produced lithium salts; comparison of cathode materials sintered with pristine and recovered lithium carbonate; characterization of recovered and pristine BTCA; process diagrams, experimental flowcharts, and equipment usage for BTCA/OA/LTA and conventional hydrometallurgical lithium recovery; ICP-OES analysis of feedstock composition and product purity; acid-dosage calculations (PDF)
