# Supplementary material for: Toward Sustainable Lithium Recovery: A Universal Hydrothermal Approach for Lithium Extraction
Source: ACS Appl Mater Interfaces. 2026 Apr 10;18(15):22057–70. doi: 10.1021/acsami.6c02211 (PMC13107369; doi:10.1021/acsami.6c02211)
Supplement: Supplementary file 1 [file am6c02211_si_001.pdf]

Supporting Information

Toward Sustainable Lithium Recovery: A Universal Hydrothermal Approach for Lithium Extraction

Zexin Wang†[1], Jiahui Hou†[1], Zifei Meng†, Jinzhao Fu†, Zeyi Yao†, Zhenzhen Yang‡, Yan Wang†*

† Department of Mechanical and Materials Engineering, Worcester Polytechnic Institute, 100 Institute Road, Worcester, MA 01609, USA

‡ Chemical Science & Engineering, Argonne National Laboratory, 9700 S Cass Ave, Lemont, IL 60439, USA

[1] These authors contributed equally.

* Corresponding: yanwang@wpi.edu

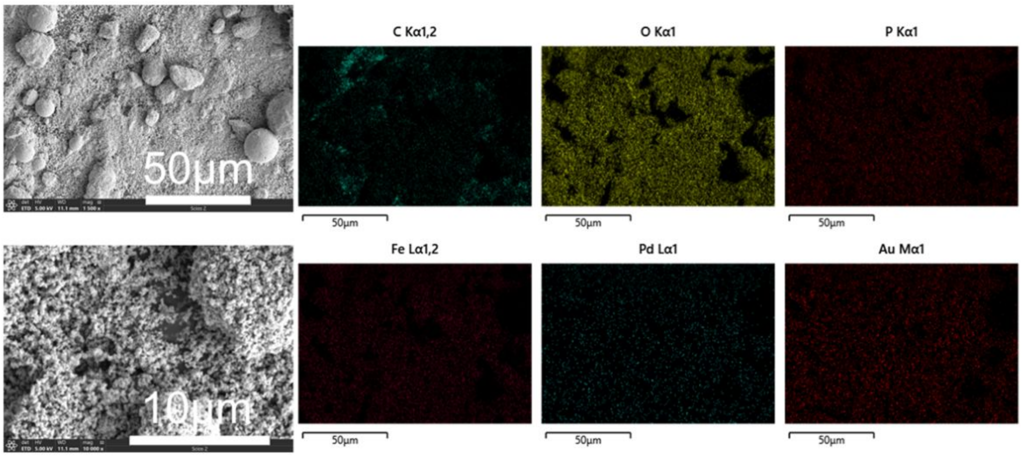

Figure S1 SEM and EDS-mapping of the pristine LFP.

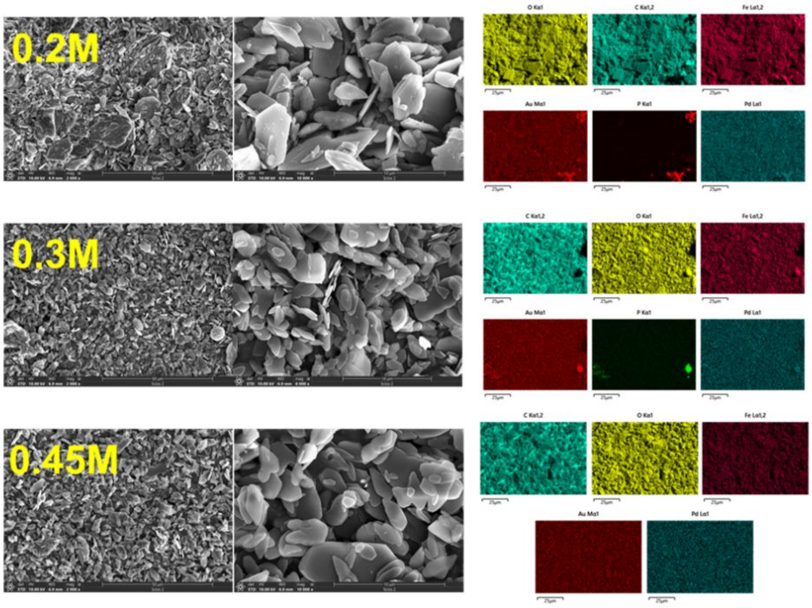

Figure S2 SEM and EDS-mapping of the leachate with the different concentrations of BTCA.

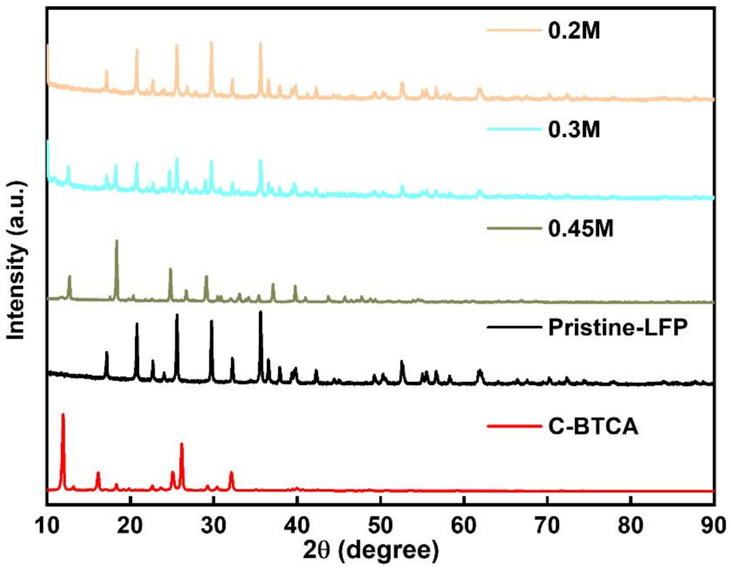

Figure S3 XRD pattern of the leachate with the different concentrations of BTCA and pristine LFP.

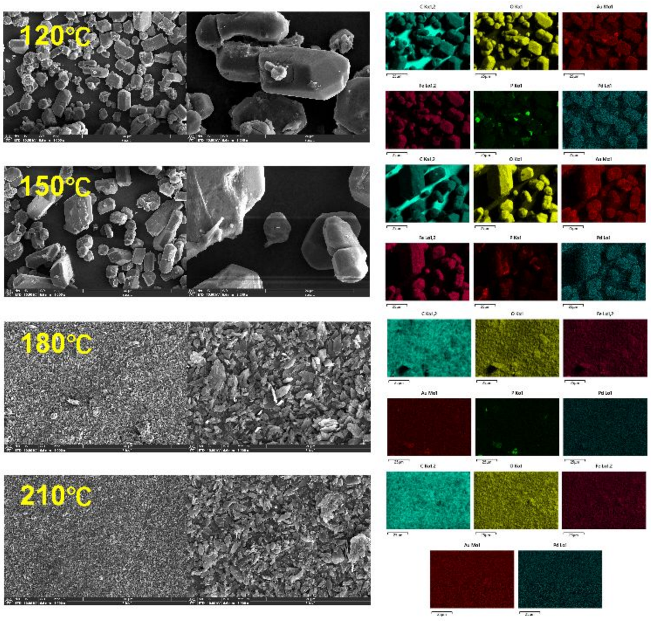

Figure S4 SEM and EDS-mapping images of the leachate with the different temperatures of BTCA.

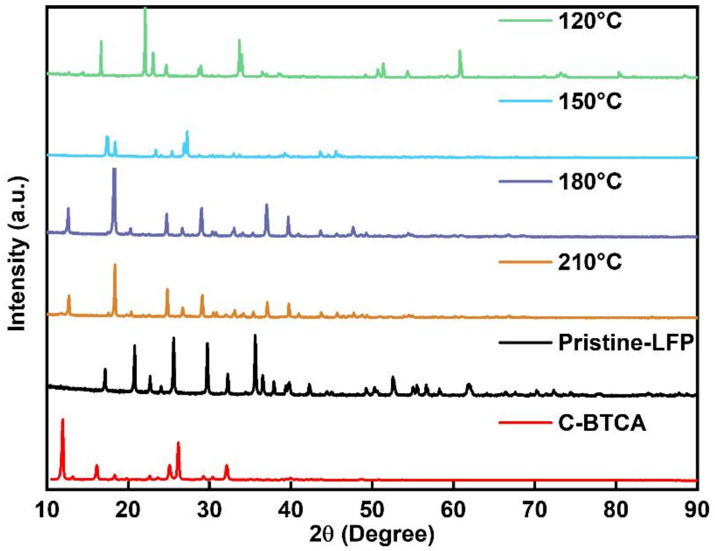

Figure S5 XRD pattern of the leachate with the different temperatures of BTCA.

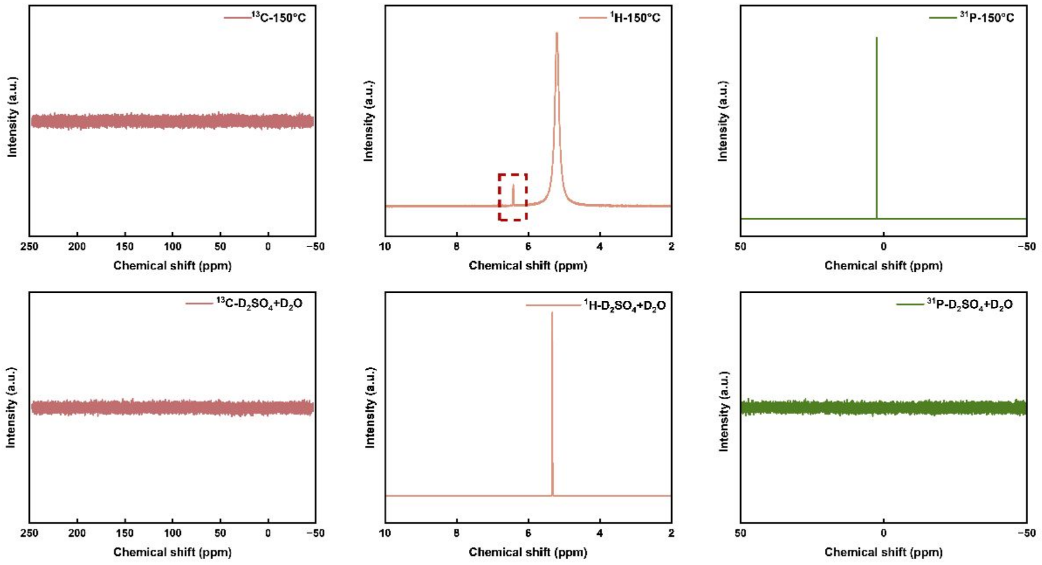

Figure S6 ^{13}C, ^{1}H, and ^{31}P NMR for residue powder after 150°C reaction and pristine BTCA powder.

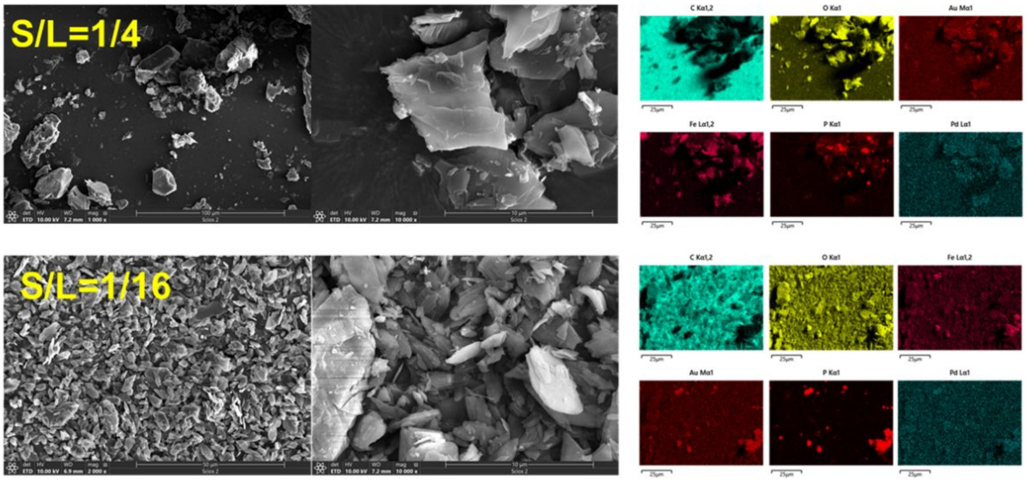

Figure S7 SEM and EDS-mapping images of the leachate with the different solid-to-liquid ratios of BTCA.

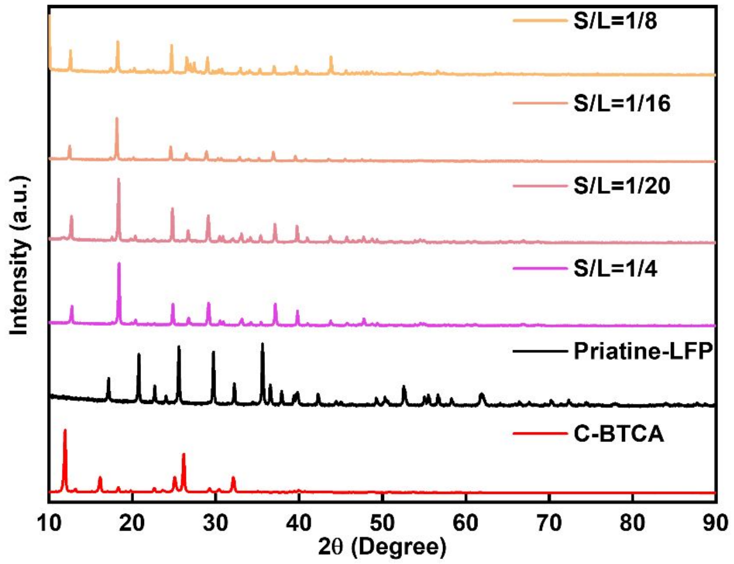

Figure S8 XRD pattern of the leachate with the different solid-to-liquid ratios of BTCA.

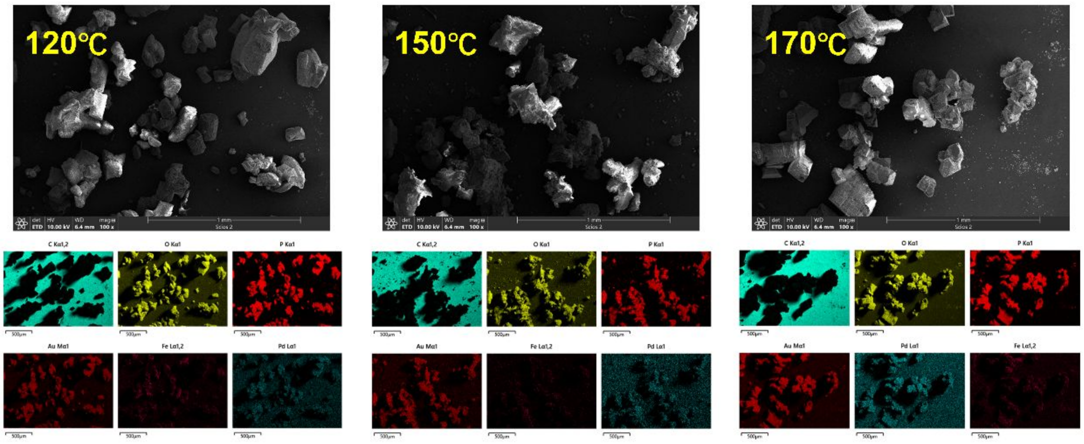

Figure S9 SEM and EDS-mapping images of the leachate of the reaction at different temperatures.

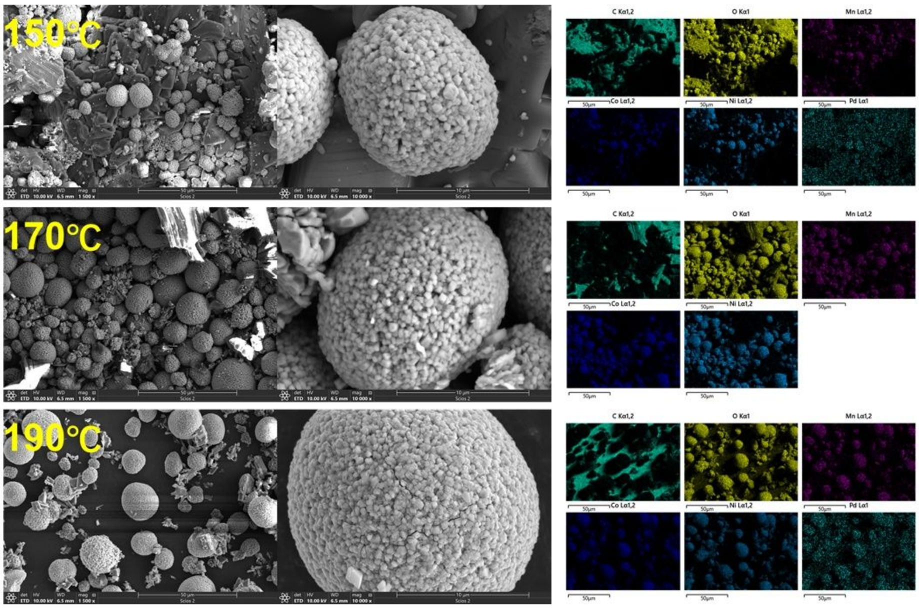

Figure S10 SEM and EDS-mapping images of the leachate with the different temperatures of BTCA.

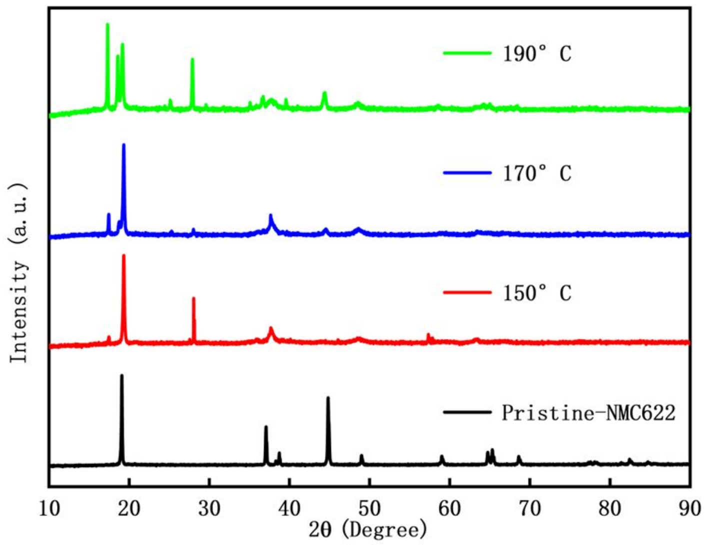

Figure S11 XRD pattern of the leachate with the different temperatures of BTCA.

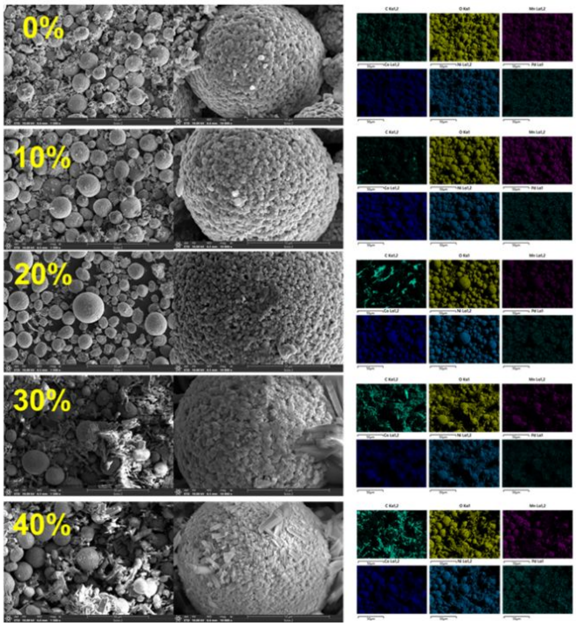

Figure S12 SEM and EDS-mapping of the leachate with the different concentrations of BTCA.

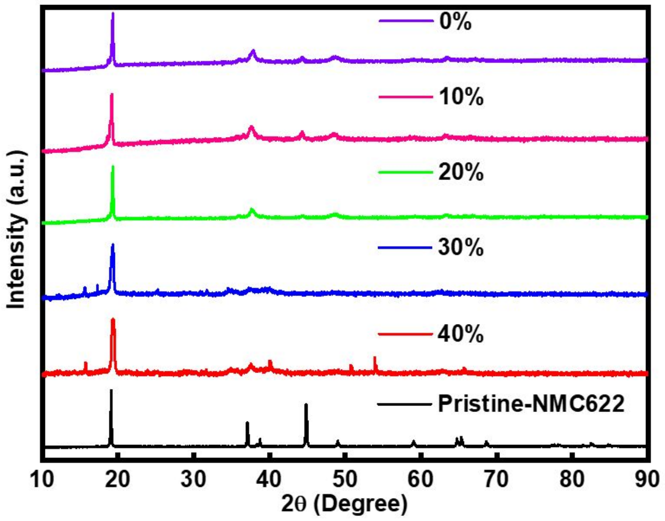

Figure S13 XRD pattern of the leachate with the different concentrations of BTCA.

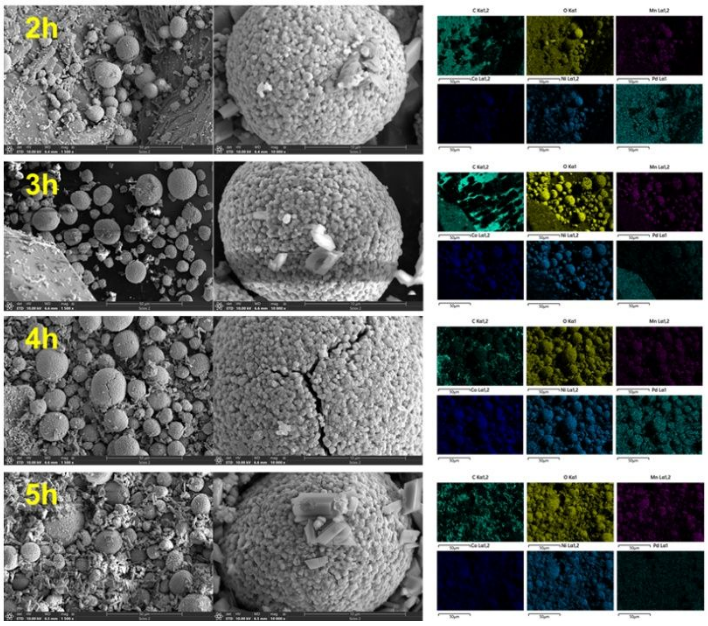

Figure S14 SEM and EDS-mapping of the leachate with the different reaction times of BTCA.

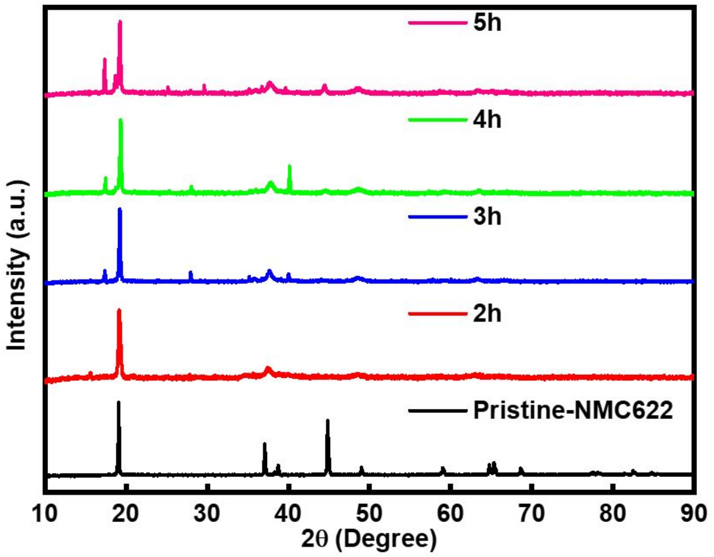

Figure S15 XRD pattern of the leachate with the different reaction times of BTCA.

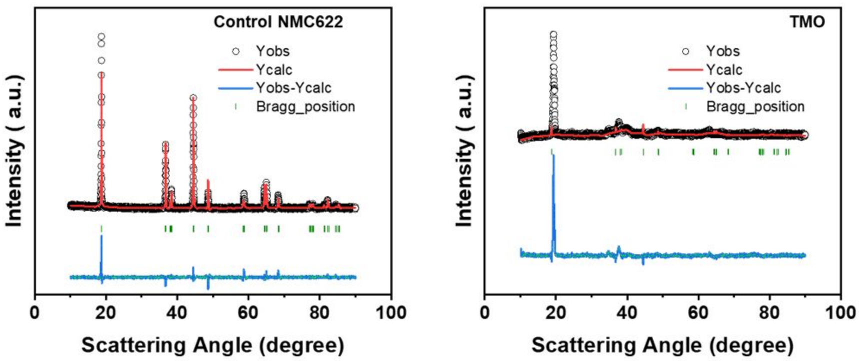

Figure S16 XRD refinement for C-NMC622 and R-TMO

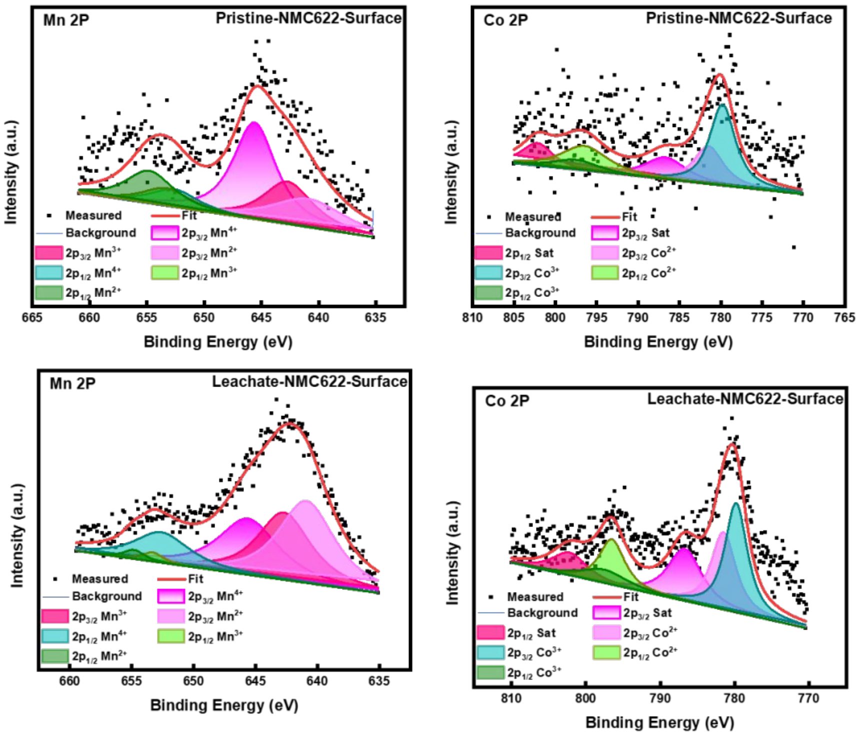

Figure S17 XPS spectrum for Mn 2p and Co 2p for pristine NMC622 and residue powder post extraction reaction (leached-NMC622).

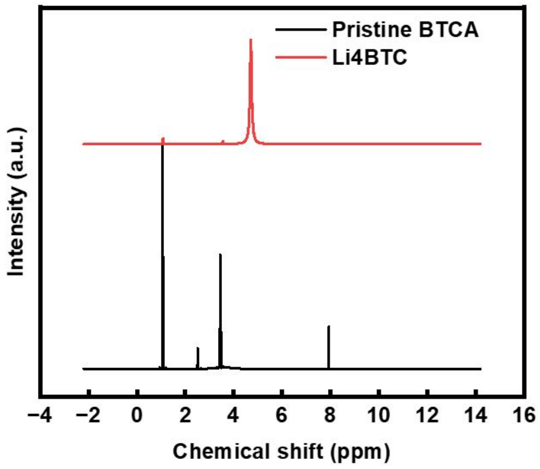

Figure S18 NMR analysis of benzene-1,2,4,5-tetracarboxylate (Li4BTC)

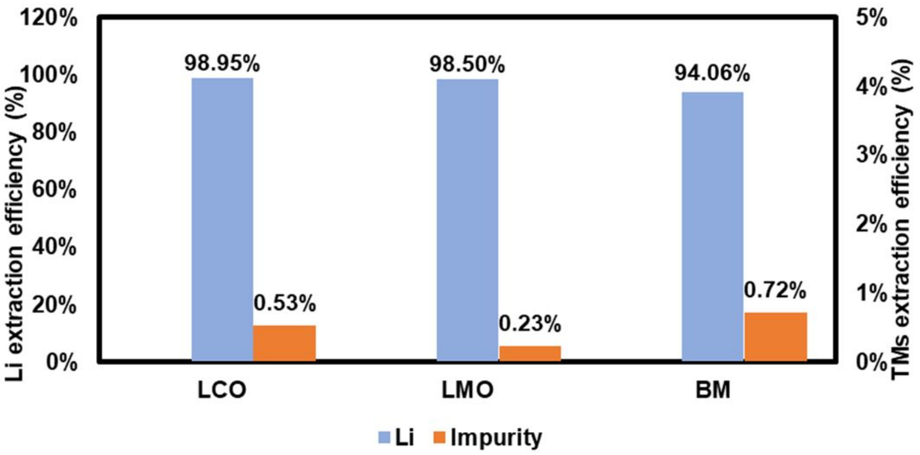

Figure S19 Lithium and transition metals leaching efficiencies of the LCO, LMO, and BM with BTCA.

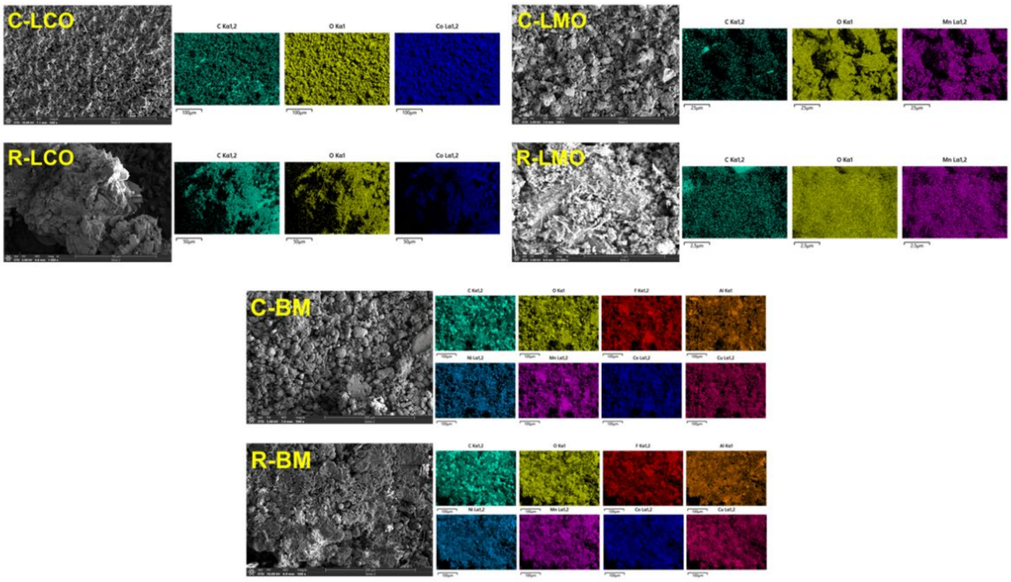

Figure S20 SEM images and EDS mapping for pristine LCO, extracted LCO, pristine LMO, extracted LMO, pristine black mass, and extracted black mass.

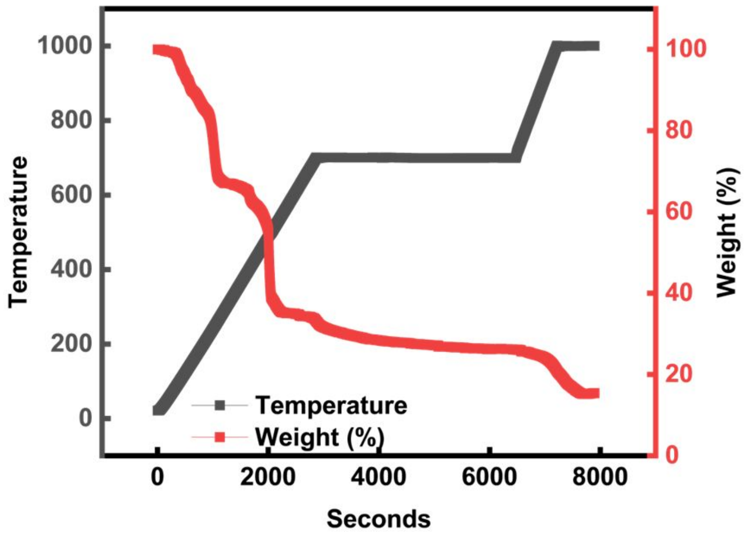

Figure S21 Thermogravimetric analysis (TGA) of the Li_4BTC

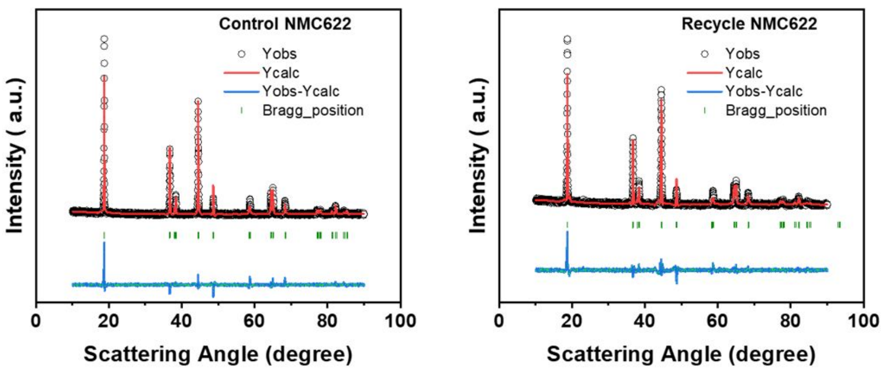

Figure S22 XRD refinement for C-NMC622 and R-NMC622

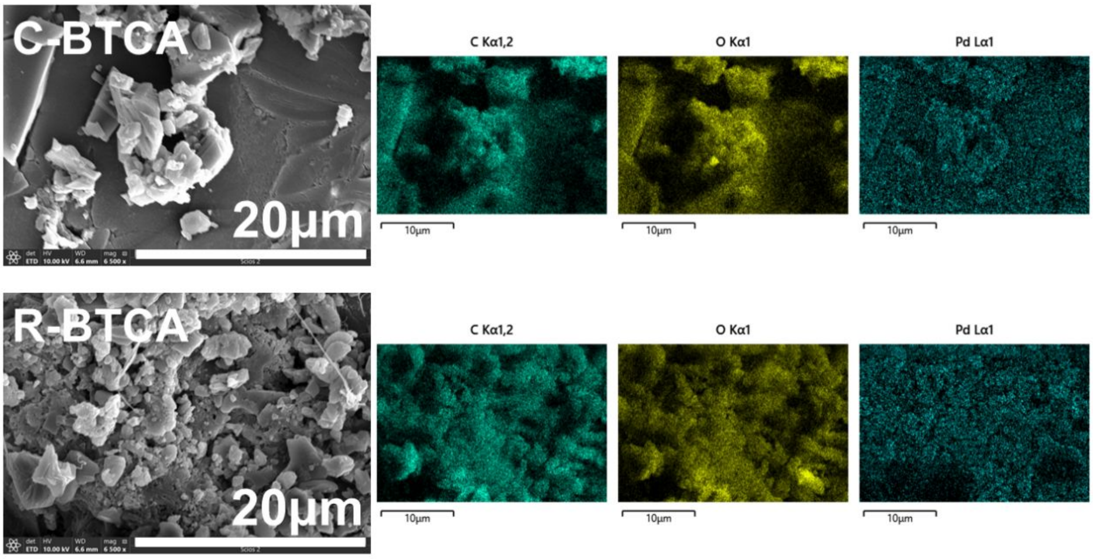

Figure S23 SEM and EDS-mapping images of the C-BTCA and R-BTCA

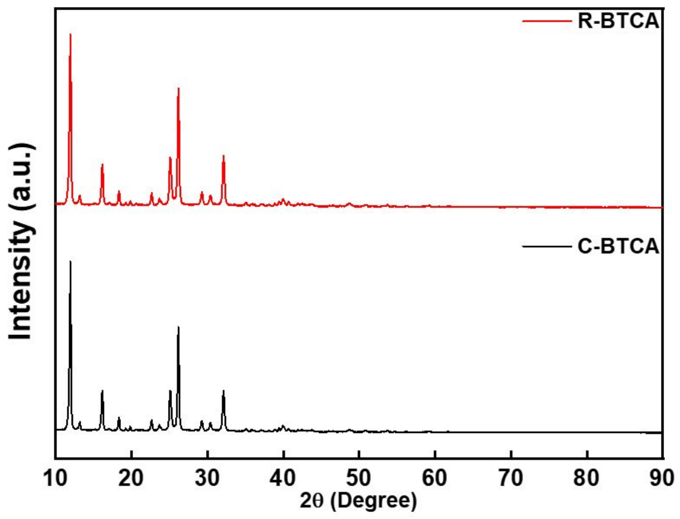

Figure S24 XRD pattern of the R-BTCA and C-BTCA

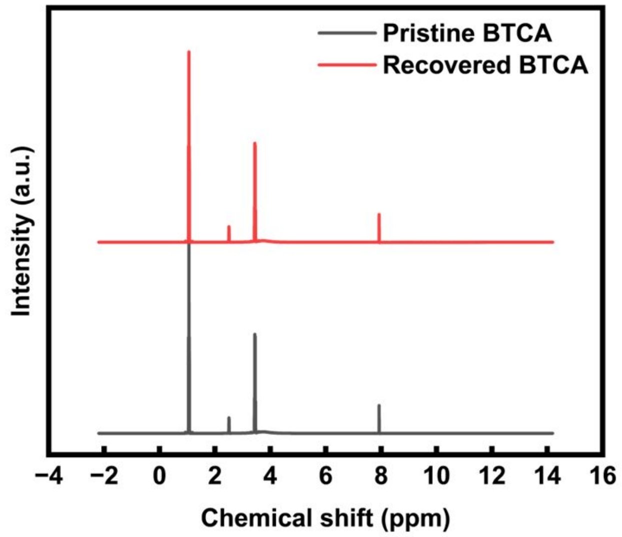

Figure S25 NMR analysis of P-BTCA and R-BTCA

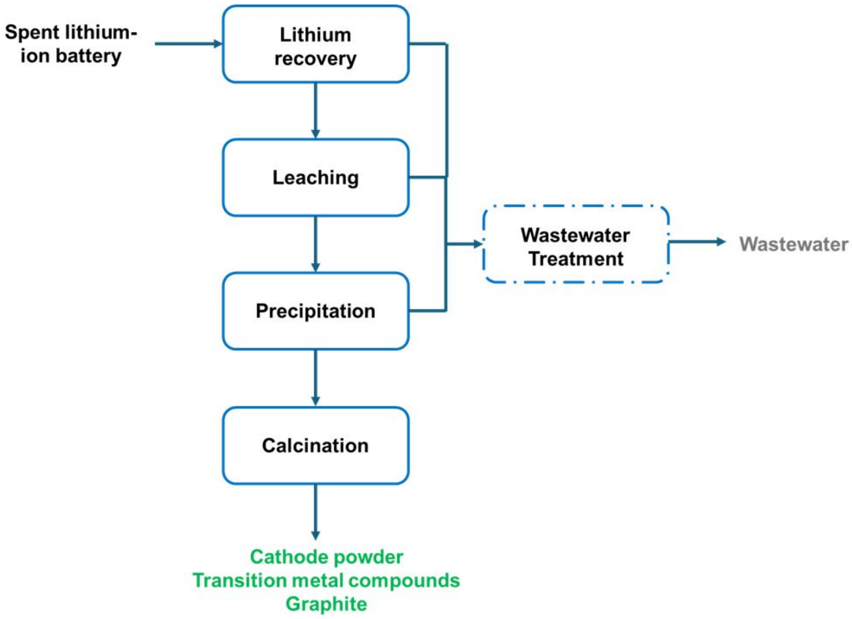

Figure S26 Process diagram of the BTCA-lithium recovery process. Solid boxes denote common unit operations; dashed box denotes optional unit operation; green denotes products; grey denotes wastes.

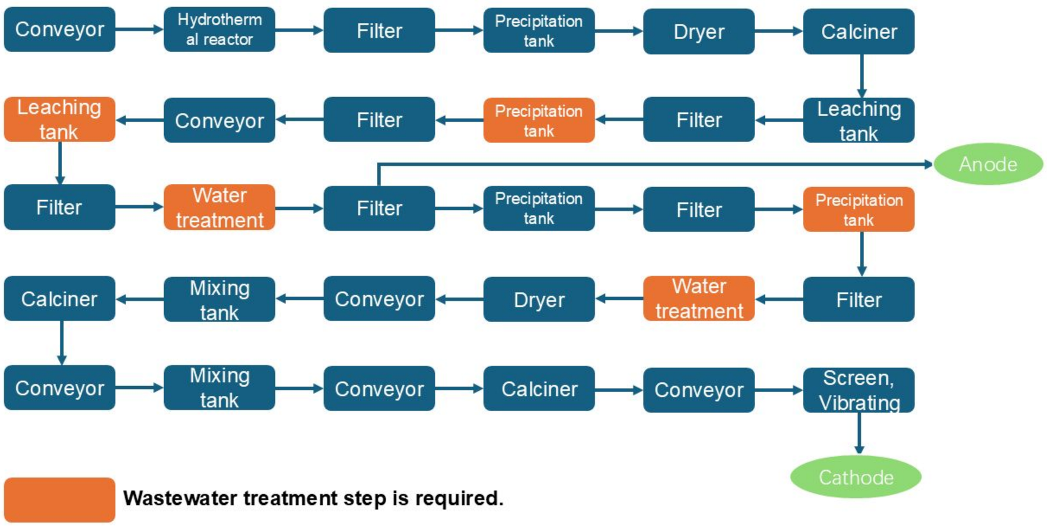

Figure S27 Detailed process and equipment usage for the BTCA-lithium recovery process.

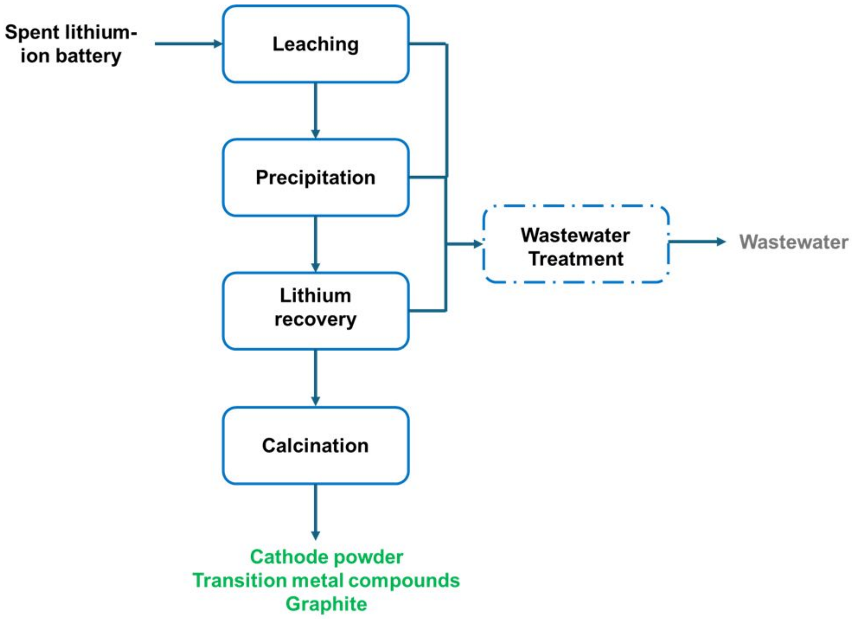

Figure S28 Process diagram of the traditional lithium recovery process. Solid boxes denote common unit operations; dashed box denotes optional unit operation; green denotes products; grey denotes wastes.

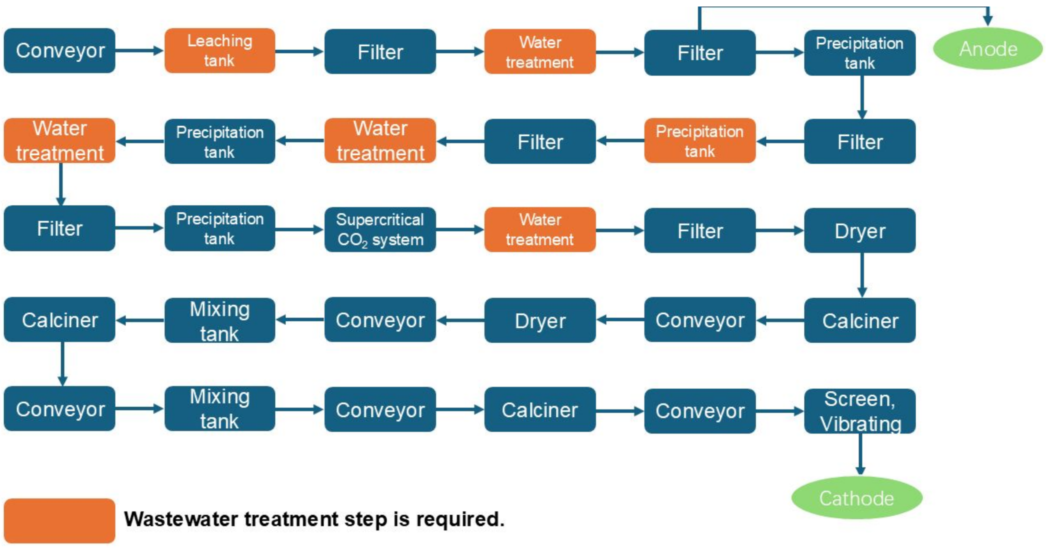

Figure S29 Detailed process and equipment usage for the traditional lithium recovery process.

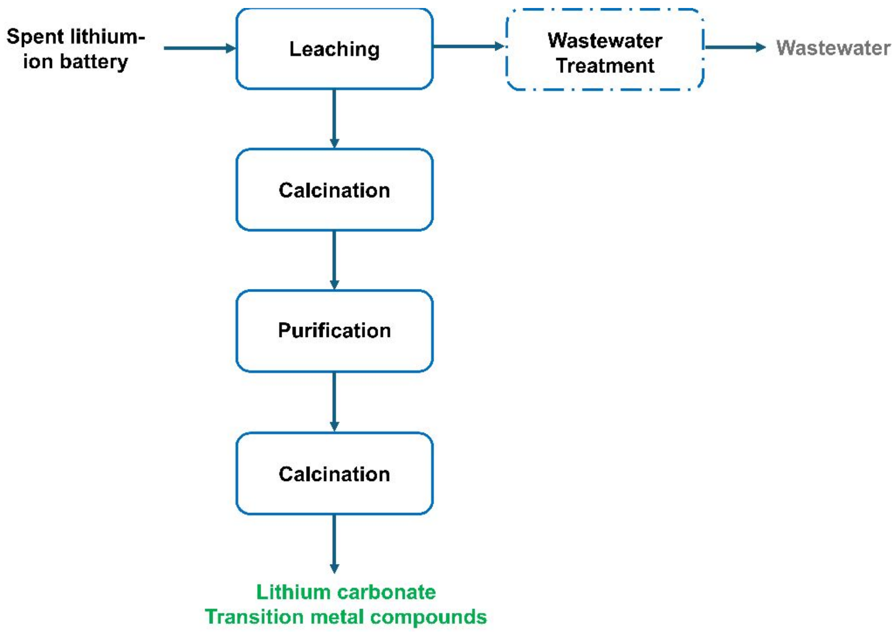

Figure S30 Process diagram of the OA-lithium recovery process. Solid boxes denote common unit operations; dashed box denotes optional unit operation; green denotes products; grey denotes wastes.

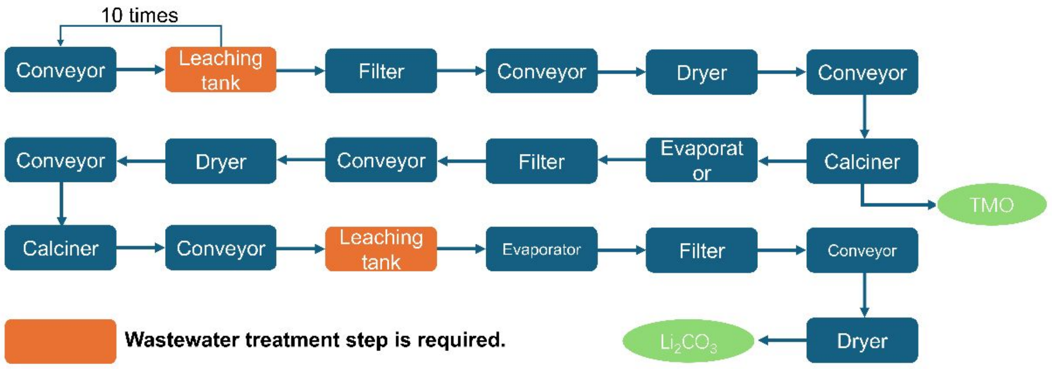

Figure S31 Detailed process and equipment usage for the OA-lithium recovery process.

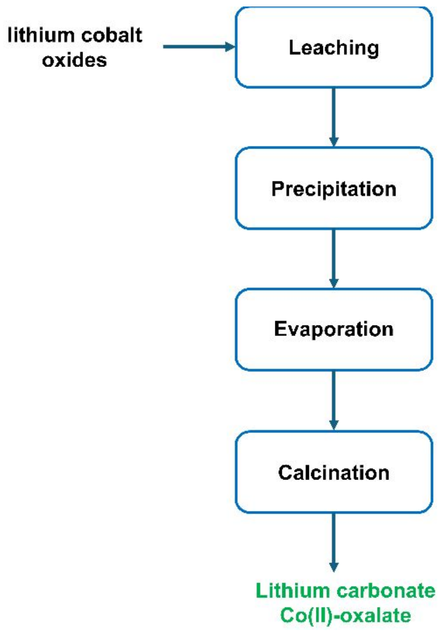

Figure S32 Process diagram of the LTA-lithium recovery process. Solid boxes denote common unit operations; green denotes products; grey denotes wastes.

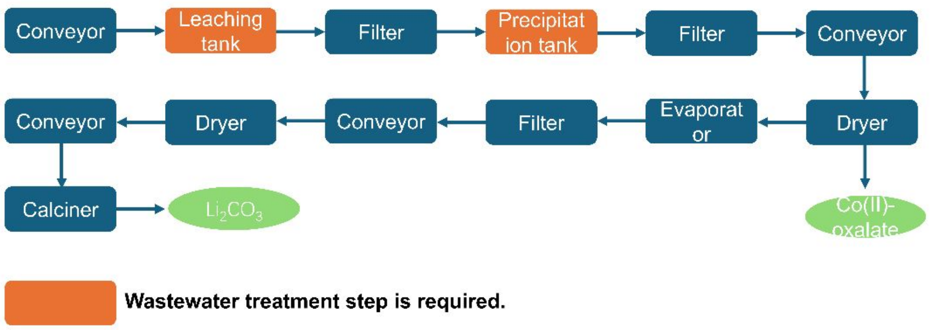

Figure S33 Detailed process and equipment usage for the LTA-lithium recovery process.

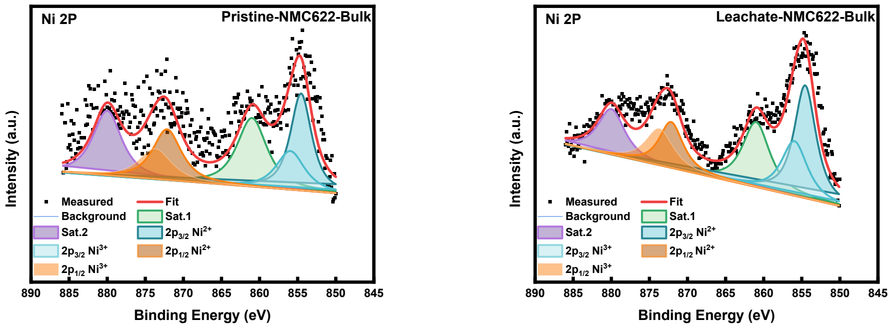

Figure S34 Bulk XPS test results for pristine and leached NMC622 cathode powder.

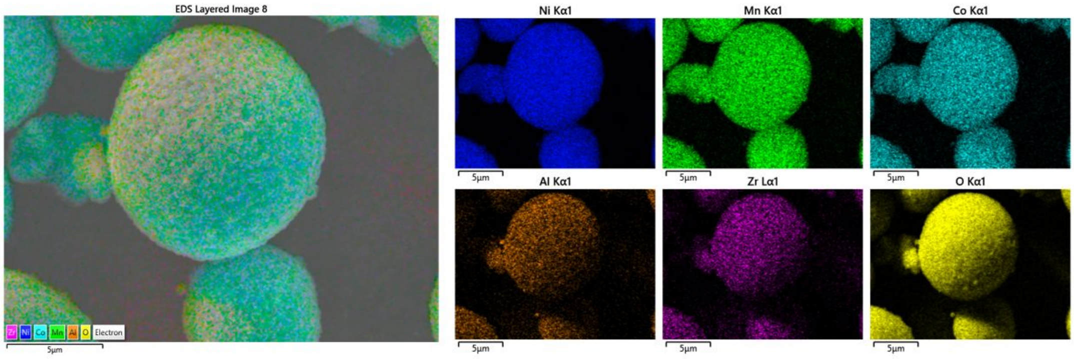

Figure S35 EDS-mapping for V-NMC622.

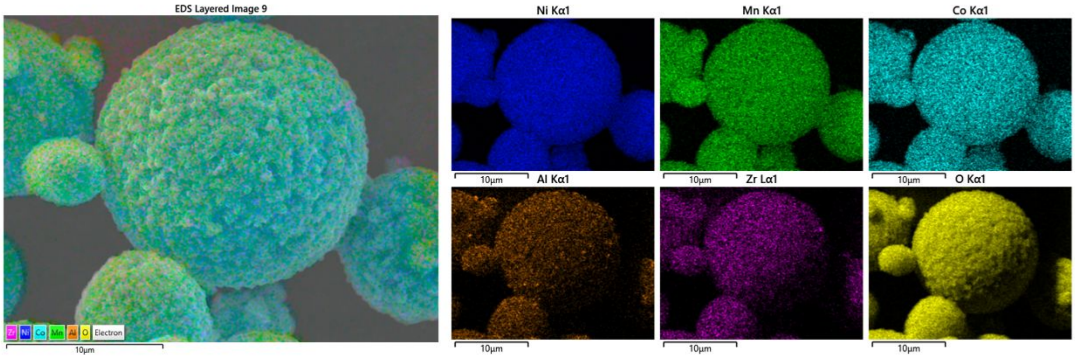

Figure S36 EDS-mapping for R-NMC622.

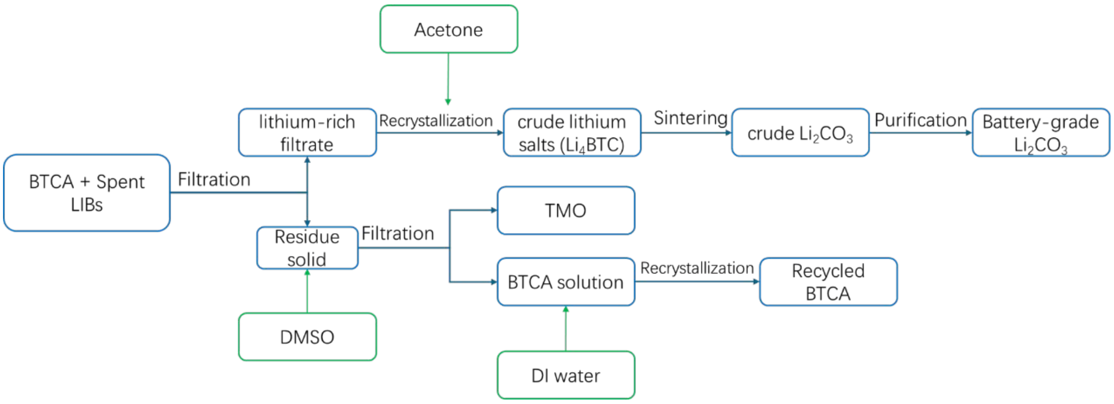

Figure S37 flow chart of the experiment.

Table S1 Summarized stoichiometric elemental ratio obtained from ICP-OES analysis for different raw materials

Sample ID	Li	Ni	Mn	Co	Fe	P
NMC622	1.02	0.6	0.2	0.2	-	-
LMO	1.01	-	2	-	-	-
LCO	1.03	-	-	1	-	-
LFP	1.03	-	-	-	1.0	1
Black mass	0.83	0.21	0.57	0.22	0.2wt%	-

Table S2 ICP-OES analysis for recovered Li_3PO_4.

ICP test results (mg/kg)													
	**Mg**	**Al**	**Si**	**S**	**Ca**	**Ti**	**Mn**	**Fe**	**Co**	**Ni**	**Cu**	**Zn**	**Purity%**
**C-Li_3PO_4**	10	103	215	210	/	/	30	92	8	38	/	38	**99.87%**
**R-Li_3PO_4**	28	132	392	216	/	/	/	105	/	/	/	18	**99.95%**

Table S3 Detailed calculation for excess of BTCA.

Excess of BTCA	Base BTCA
0%	MLi*1*0.25MWBTCA
10%	MLi*1.1*0.25MWBTCA
20%	MLi*1.2*0.25MWBTCA
30%	MLi*1.3*0.25MWBTCA
40%	MLi*1.4*0.25MWBTCA
50%	MLi*1.5*0.25MWBTCA

*MLi=mol ratio of lithium in CAM

*MWBTCA=molar weight of BTCA

Table S4 Detailed structure from XRD refinement

	a-axis	c-axis	Volume	Ni in Li layer	X^2	Rwp
Control NMC622	2.86678	14.21054	101.141	1.20	3.61	5.59
R-TMO	2.86684	14.21377	101.169	-	22.8	7.53

Table S5 ICP-OES analysis for recovered Li_2CO_3.

	ICP test results (mg/kg)												
	**Mg**	**Al**	**Si**	**S**	**Ca**	**Ti**	**Mn**	**Fe**	**Co**	**Ni**	**Cu**	**Zn**	**Purity%**
**C-Li_2CO_3**	13	87	315	110	/	/	35	102	38	53	/	32	**99.92%**
**R-Li_2CO_3**	25	32	92	56	/	/	73	55	42	55	/	15	**99.97%**

Table S6 Detailed structure from XRD refinement.

	a-axis	c-axis	Volume	Ni in Li layer	X^2	Rwp
Control NMC622	2.86678	14.21054	101.141	1.20	3.61	5.59
Recycle NMC622	2.86741	14.21805	101.240	1.20	4.35	6.13

Table S7 ICP-OES results for commercial BTCA and recovered BTCA

	ICP-OES results (mg/kg)						
	Al	Co	Cu	Fe	Li	Mn	Ni
R-BTCA	0.009	0	0	0	0	0	0
C-BTCA	0	0	0	0	0	0	0

Table S8 ICP-OES results for V-NMC622 and R-NMC622

	ICP-OES results (mg/kg)					
	Al	Co	Ni	Mn	Li	Zr
V-NMC622	0.34wt%	0.2	0.6	0.2	1.02	0.2mol%
R-NMC622	0.35wt%	0.2	0.6	0.2	1.02	0.2mol%
